# DNA nicks induce mutational signatures associated with *BRCA1* deficiency

**DOI:** 10.1038/s41467-022-32011-x

**Published:** 2022-07-25

**Authors:** Yi-Li Feng, Qian Liu, Ruo-Dan Chen, Si-Cheng Liu, Zhi-Cheng Huang, Kun-Ming Liu, Xiao-Ying Yang, An-Yong Xie

**Affiliations:** 1 Innovation Center for Minimally Invasive Technique and Device, Department of General Surgery, Sir Run Run Shaw Hospital, Zhejiang University School of Medicine, 310019 Hangzhou, Zhejiang P. R. China; 2 Institute of Translational Medicine, Zhejiang University School of Medicine and Zhejiang University Cancer Center, 310029 Hangzhou, Zhejiang P. R. China

**Keywords:** DNA, Cancer genetics

## Abstract

Analysis of human cancer genome sequences has revealed specific mutational signatures associated with *BRCA1*-deficient tumors, but the underlying mechanisms remain poorly understood. Here, we show that one-ended DNA double strand breaks (DSBs) converted from CRISPR/Cas9-induced nicks by DNA replication, not two-ended DSBs, cause more characteristic chromosomal aberrations and micronuclei in *Brca1*-deficient cells than in wild-type cells. BRCA1 is required for efficient homologous recombination of these nick-converted DSBs and suppresses bias towards long tract gene conversion and tandem duplication (TD) mediated by two-round strand invasion in a replication strand asymmetry. However, aberrant repair of these nick-converted one-ended DSBs, not that of two-ended DSBs in *Brca1*-deficient cells, generates mutational signatures such as small indels with microhomology (MH) at the junctions, translocations and small MH-mediated TDs, resembling those in *BRCA1*-deficient tumors. These results suggest a major contribution of DNA nicks to mutational signatures associated with *BRCA1* deficiency in cancer and the underlying mechanisms.

## Abstract (web summary)

It remains unclear how BRCA1-associated mutational sigantures are generated in cancer. Here, the authors develop nCas9-based approaches to uncover that aberrant repair of one-ended DSBs converted from DNA nicks by replication, not that of two-ended DSBs, induces such mutational signatures.

## Introduction

Somatic mutations in human cancer genomes are generated by various mutational mechanisms involving DNA replication, damage and repair during the development of cancer. Each mechanism imprints somatic mutations with a characteristic pattern known as mutational signatures. Analysis of cancer genome sequences across cancer types has identified many distinct classes of mutational signatures. *BRCA1*-deficient cancers including breast, ovary and other cancers exhibit the SBS3 signature characterized by the uniform distribution of all base substitution types, the ID6 signature by small 11–50 bp indels with short microhomology (MH) at junctions, small ~10 kb MH-mediated TDs, and unbalanced translocations^1–7^. Because *BRCA1* deficiency severely impairs error-free homologous recombination (HR), one of two major repair pathways for DNA double-strand breaks (DSBs), *BRCA1*-deficient cells become more dependent upon alternative pathways, particularly error-prone non-homologous end joining (NHEJ), for the repair of DSBs^8,9^. It is tempting to speculate some of these alternative repair pathways may generate mutational signatures associated with *BRCA1* deficiency. In fact, several studies have implicated polymerase theta-mediated end joining (TMEJ) in the generation of mutational signatures in *BRCA1*-deficient tumors^10–12^. However, as BRCA1 participates in different steps of HR, which repairs DSBs from various sources, and is also required for replication fork protection, it is poorly understood how individual mutational signature is developed in *BRCA1*-deficient tumors^8,9,13^.

DSBs appear in two general forms: two-ended and one-ended. The HR function of BRCA1 is well recognized in repair of two-ended DSBs. In early step of HR, BRCA1 counteracts 53BP1 to facilitate end resection^14^. By interaction with PALB2 and recruitment of BRCA2, BRCA1 also promotes RAD51 filament formation, a critical step for strand invasion into a homologous DNA during HR^8,15,16^. HR is mostly completed by gene conversion, which typically extends less than 100 bp for short-tract gene conversion (STGC) but can, albeit infrequently, extend to several kilobases for aberrant long-tract gene conversion (LTGC)^17^. BRCA1 suppresses LTGC in HR repair of two-ended DSBs at least in part due to second-end resection that provides 3’ single-stranded DNA (ssDNA) for strand annealing in timely termination of STGC^18^. Compared to two-ended DSBs that rarely occur spontaneously, collapsed or stalled replication forks often generate one-ended DSBs as the primary substrate for HR in mammalian cells^19,20^. Unlike two-ended DSBs that can be efficiently repaired by end rejoining, one-ended DSBs are more dependent on HR for repair due to lack of the second end and often trigger break-induced replication (BIR) in yeast and possibly in mammalian cells^21,22^. In fact, by using *Escherichia coli* Tus–Ter replication fork barriers to trigger site-specific replication fork stalling at a mammalian chromosome, a study found that BRCA1 suppresses LTGC, a potentially BIR analogue in mammalian cells, at collapsed replication forks where one-ended DSBs can arise^23^. In addition, replication fork stalling promotes the formation of small ~10 kb MH-mediated TDs in *Brca1*-deficient mouse embryonic stem cells (mESC)^24^. However, given that single-strand breaks (SSBs) including nicks account for about 75% of endogenous DNA lesions detected daily in mammalian cells, it is likely that some of these SSBs could collapse replication forks and produce one-ended DSBs as a principal endogenous source^25–29^. However, while BRCA1 is also expected to participate in HR repair of such one-ended DSBs, much has yet to be learnt about the consequences of BRCA1 dysfunction.

In order to study the role of BRCA1 in the repair of DSBs converted from SSBs by DNA replication and the contribution of this role to mutational signatures in *BRCA1*-deficient cells, we established a cellular assay system where a site-specific nick can be induced by *Streptococcus pyogenes* Cas9 (*Sp*Cas9) nickases (nCas9)^30,31^ and subsequently converted into a one-ended DSB by DNA replication in mammalian cells. Using this system, we demonstrated that *BRCA1* was required for efficient HR of replication-dependent DSBs converted from nicks but suppressed LTGC bias during this HR repair. We also found that DNA nicks, not two-ended DSBs, induced mutational signatures such as the small indel signature, translocations, and MH-mediated TDs (~10 kb) in *BRCA1*-deficient mESC, resembling those in *BRCA1*-deficient tumors. In addition, nick-induced LTGC bias and repeat-directed TDs generated by two-round strand invasions were promoted in a replication strand bias in *BRCA1*-deficient cells. Our data suggest that DNA nicks coupled with DNA replication, not two-ended DSBs, could be a major inducer to characteristic mutational signatures in *BRCA1*-deficient tumors.

## Results

### nCas9-induced nicks are converted into one-ended DSBs by DNA replication

**Fig. 1:**
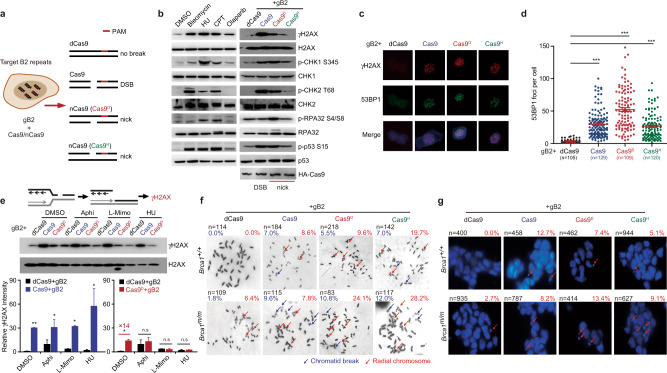
Nicks induce increased chromosomal aberrations in *Brca1*-deficient cells. **a** Schematic of DSB and nick induction at short interspersed nuclear element (SINE) B2 repeats by SpCas9 nucleases. Cas9^D^: Cas9 D10A; Cas9^H^: Cas9 H840A. The PAM is highlighted by red line. **b** Immunoblot of mESC WCEs after 2-h treatment with DNA damage-inducing agents or 24 h after transfection with expression plasmids for dCas9-gB2, Cas9-gB2, Cas9^D^-gB2, and Cas9^H^-gB2. Transfection efficiencies were similar at about 70%. **c** Representative images of 53BP1 and γH2AX focus formation in mESC at 24 h after transfection with expression plasmids for dCas9-gB2, Cas9-gB2, Cas9^D^-gB2 and Cas9^H^-gB2 as indicated. **d** Quantification of 53BP1 foci in **c**. Each dot represents the number of 53BP1 foci in each cell. **e** Immunoblot of mESC WCEs for γH2AX induction at 24 h after transfection with dCas9-gB2, Cas9-gB2, Cas9^D^-gB2, and Cas9^H^-gB2 together with replication inhibitor treatment. Top: Schematic of possible DSB induction and γH2AX generation. Middle: Immunoblot of WCEs with anti-γH2AX and anti-H2AX antibodies. Bottom: Quantification of γH2AX intensity by Image J. **f** Representative images of chromosomal aberrations in both *Brca1*^*+/+*^ and *Brca1*^*m/m*^ cells at 24 h after transfection with expression plasmids for dCas9-gB2, Cas9-gB2, Cas9^D^-gB2 and Cas9^H^-gB2 as indicated. Transfection efficiencies were similar at 70%. The number of metaphases from two independent experiments and the percentage of metaphases containing chromatid breaks and radial chromosomes are indicated. Chromatid breaks and radical chromosomes are marked by blue and red arrows, respectively. **g** Representative images of micronuclei in both *Brca1*^*+/+*^ and *Brca1*^*m/m*^ cells transfected with expression plasmids for dCas9-gB2, Cas9-gB2, Cas9^D^-gB2, and Cas9^H^-gB2. Transfection efficiencies were similar at 70%. Arrows indicate micronuclei. n denotes the number of cells from three independent experiments and the percentage of cells with micronuclei are also indicated in red. Columns indicate the mean ± S.E.M of at least three independent experiments, each in triplicates. The P value is determined by two-tailed Student’s *t* testing (**e**). Unpaired *t* test with Welch’s correction is performed to compare the 53BP1 foci per cell between two groups in (**d**). n.s, *P* > 0.05; ^*^P < 0.05; ^**^P < 0.01; ^***^P < 0.001.

We first used Cas9 or nCas9 (D10A: Cas9^D^; H840A: Cas9^H^), together with gB2, an sgRNA targeting short interspersed nuclear element B2 repeats, which are present in ~350,000 copies in the mouse genome^32^, to induce a number of DSBs or nicks in mouse cells, respectively (Fig. 1a). mESC transfected with nCas9-gB2 or Cas9-gB2 proliferated much slower (Supplementary Fig. 1a). Phosphorylation of Chk1 S345, Chk2 T68, p53 S15 and H2AX S139 (to form “γH2AX”) was stimulated in mESC by Cas9-gB2 whereas the RPA32 S4/S8 phosphorylation remained unchanged at a basal level (Fig. 1b). Likewise, both Cas9^D^ and Cas9^H^ induced phosphorylation of H2AX, Chk1, Chk2, and p53 (Fig. 1b); however, Cas9^D^ induced more phosphorylation of Chk1 and RPA32 than Cas9 (Fig. 1b). The difference in this response between Cas9 and Cas9^D^ appears to parallel that between bleomycin and hydroxyurea (HU) or camptothecin (CPT) (Fig. 1b), indicating that Cas9^D^-induced DNA damage response (DDR) may be associated with DNA replication. Likely due to the stronger nicking activity of the HNH domain^33^, the DDR induced by Cas9^D^ is much stronger than that by Cas9^H^.

We also examined Cas9- or nCas9-induced focus formation of γH2AX and 53BP1, both of which serve as a DSB marker. Both Cas9 and nCas9-induced co-localized formation of γH2AX and 53BP1 foci as did bleomycin, HU and CPT (Fig. 1c and Supplementary Fig. 1b, c). Cas9^D^ induced even more focus formation than Cas9 in mESC, not in NIH3T3, suggesting possibly more efficient conversion of nicks into one-ended DSBs by DNA replication in mESC (Fig. 1d and Supplementary Fig. 1b, c). To further confirm that this conversion is coupled with DNA replication, we treated mESC with DNA replication inhibitors and found that this treatment had little effect on relative intensity of Cas9-induced γH2AX but abolished Cas9^D^-induced γH2AX (Fig. 1e). Together, these results suggest that nCas9-induced nicks could be efficiently converted into one-ended DSBs by DNA replication.

### Nicks, not two-ended DSBs, induce *BRCA1*-linked chromosomal aberrations

Using paired Cas9-sgRNA approach^34^, we generated *Brca1*-deficient (*Brca1*^*m/m*^) mESC in which mutated *Brca1* gene encodes unstable BRCA1 lacking the C-terminal BRCT repeats (Supplementary Fig. 1d, e)^35^. Cas9-gB2 and nCas9-gB2 were transfected to induce a number of two-ended DSBs and nicks in isogenic *Brca1*^+/+^ and *Brca1*^m/m^ mESC (Supplementary Fig. 1f). In cells expressing the dCas9-sgRNA negative control, the chromosome abnormalities spontaneously arose only in *Brca1*-deficient cells (Fig. 1f and Supplementary Fig. 1g, h). It is possible that DNA-bound dCas9 may act as a barrier to transcription or DNA replication at many B2 target sites, thus causing DNA damage^36–39^. Either Cas9-gB2 or nCas9-gB2 induced a high level of abnormal metaphases in both *Brca1*^+/+^ and *Brca1*^m/m^ cells (Fig. 1f and Supplementary Fig. 1g, h). However, the level of chromosomal aberrations induced by Cas9-gB2 was similar or slightly different between *Brca1*^+/+^ and *Brca1*^m/m^ mESC. In contrast, chromosomal aberrations induced by Cas9^D^- and Cas9^H^-gB2 were much more severe in *Brca1*^m/m^ mESC (Fig. 1f and Supplementary Fig. 1g, h), suggesting that nicks (or by extension one-ended DSBs) and aberrant repair of these DNA lesions may be a major source for spontaneous chromosomal aberrations associated with *BRCA1* deficiency. It is however unclear why Cas9^D^-gB2 appeared to induce a lower level of chromosomal aberrations than Cas9^H^-gB2 (Fig. 1f), although Cas9^D^ caused a stronger DNA damage response due to its better cutting activity.

*Brca1*-deficient MEFs have significant micronuclei formation^40^. We thus analyzed micronuclei formation induced by Cas9-gB2 or nCas9-gB2. In cells expressing dCas9-gB2, 2.7% *Brca1*-deficient cells exhibited micronuclei formation but no *Brca1*-proficient cells did (Fig. 1g and Supplementary Fig. 1i). Unexpectedly, micronuclei formation in *Brca1*^m/m^ cells was less frequent than in *Brca1*^+/+^ cells after transfection with Cas9-gB2 (Fig. 1g and Supplementary Fig. 1i). In contrast, micronuclei formation induced by Cas9^D^- and Cas9^H^-gB2 was more extensive in *Brca1*^m/m^ mESC than in *Brca1*^+/+^ cells (Fig. 1g and Supplementary Fig. 1i), suggesting that one-ended DSBs and aberrant repair of these DNA lesions may contribute to spontaneous micronuclei formation detected in *Brca1*-deficient cells, not in normal cells.

### HR repair of nCas9-induced nicks is coupled with DNA replication

**Fig. 2:**
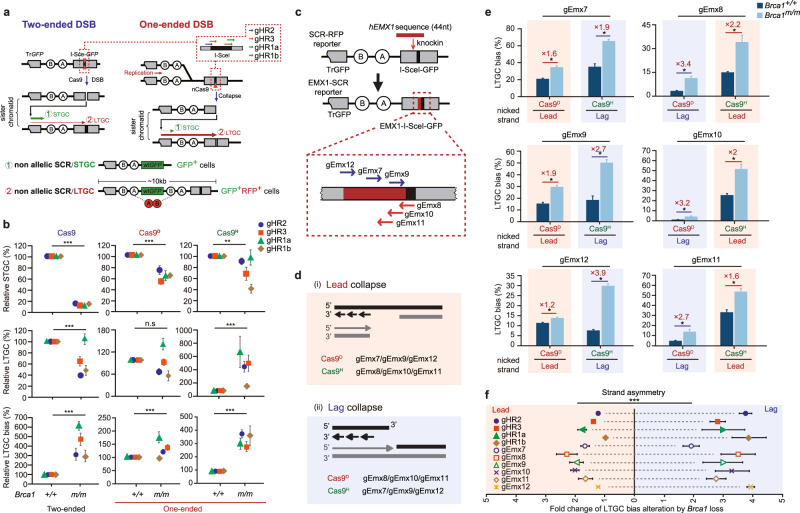
BRCA1 suppresses nick-induced LTGC bias in a strand-asymmetric manner. **a** Schematic of the SCR-RFP reporter and HR repair products induced by Cas9 and nCas9. Cas9 induces two-ended DSBs whereas nCas9-induced nicks could be converted into one-ended DSBs by DNA replication. STGC and LTGC in non-allelic SCR induced by Cas9 or nCas9 generate GFP^+^RFP^−^ cells and GFP^+^RFP^+^ cells with a TD span size at ~10 kb as indicated, respectively. Only one direction of one-ended DSBs is expected to result in GFP^+^RFP^+^ cells and the direction of DNA replication generating such one-ended DSBs is indicated. Gray boxes: *TrGFP* and *I-Sce-GFP*; green box: wt*GFP*; circles A and B: two artificial *RFP* exons. Four sgRNAs targeting the I-SceI site are denoted. **b** Relative STGC (top), LTGC (middle) and LTGC bias (bottom) induced by Cas9 or nCas9 together with each of 4 sgRNAs as indicated in isogenic *Brca1*^*+/+*^ and *Brca1*^*m/m*^ mESC. Relative STGC, LTGC and LTGC bias are derived from the original measurements in Supplementary Fig. 4c by normalizing STGC, LTGC and LTGC bias in *Brca1*^*+/+*^ cells to 100%. **c** Schematic for generation of the EMX1-SCR reporter in mESC. The EMX1-SCR reporter was generated by knock-in of a 44-nt h*EMX1* sequence as indicated at 5’ of the I-SceI site of the SCR-RFP reporter in mESC. Among 6 sgRNAs targeting the EMX1-I-SceI site, gEmx7, gEmx9 and gEmx12 target the PAM on the Watson strand, and gEmx8, gEmx10 and gEmx11 on the Crick strand as indicated. **d** Schematic for generation of one-ended DSBs by collision of nCas9-induced nicks with the leading strand or the lagging strand of DNA replication. Collapse of DNA replication forks by collision with the leading strand or the lagging strand generates one-ended DSBs and is respectively termed lead collapse (**i**) or lag collapse (**ii**). nCas9-sgRNAs that cause lead collapse and lag collapse are denoted. **e** Alteration of LTGC bias between *Brca1*^*+/+*^ and *Brca1*^*m/m*^ cells. The replication strand that encounters nCas9-induced nicks, potentially leading to production of GFP^+^RFP^+^ cells, is indicated. The fold of increase in LTGC bias between *Brca1*^*+/+*^ and *Brca1*^*m/m*^ cells is stated above each pair of columns for each indicated nCas9-sgRNA. **f** Combined analysis of strand asymmetry in alteration of LTGC bias by *Brca1* deficiency. The replication strand that encounters nCas9-induced nicks, potentially leading to production of GFP^+^RFP^+^ cells as LTGC events, is indicated. Each symbol represents the mean of at least three independent experiments for one sgRNA and statistics is performed by two-tailed Student’s t testing (**b**), **f** Columns indicate the mean ± S.E.M and statistical significance is detected by two-tailed Student’s *t* testing (**e**). ^*^P < 0.05 and ^***^P < 0.001.

As HR between sister chromatids, i.e., sister chromatid recombination (SCR), is a predominant pathway in repair of one-ended DSBs at collapsed replication forks^41,42^, defects of this HR repair may be responsible for elevated chromosomal aberrations in *BRCA1*-deficient cells. We used mESC harboring a single-copy “SCR-RFP” reporter integrated at the *Rosa26* locus to characterize HR repair of one-ended DSBs (Fig. 2a and Supplementary Fig. 2a)^18,43^. In this SCR-RFP reporter containing 5’ truncated *GFP* (*TrGFP*) and *I-Sce-GFP* interrupted by insertion of an 18-bp recognition site for the rare cutting endonuclease I-SceI, site-specific two-ended DSBs induced by I-SceI or Cas9 at *I-Sce-GFP* could be repaired by non-allelic SCR that uses *TrGFP* of sister chromatid as a homologous template. STGC in this non-allelic SCR generates GFP^+^RFP^−^ cells while LTGC results in GFP^+^RFP^+^ cells by *GFP* repeat-mediated TD of the *RFP* exon B and A cassette (Fig. 2a). To study HR repair of one-ended DSBs, a nick could be induced around the I-SceI site of the reporter and converted into a one-ended DSB by collision with either the leftward or the rightward DNA replication fork (Fig. 2a). One-ended DSBs generated from either direction may invade *TrGFP* to start non-allelic SCR, which is terminated by the homologous second end generated by the converging replication fork via second-end capture or homologous annealing, leading to STGC or LTGC. While GFP^+^RFP^−^ cells are produced by STGC, only one-ended DSBs from the rightward fork can normally engage LTGC that duplicates the *RFP* exon B and A cassette, generating GFP^+^RFP^+^ cells (Fig. 2a).

After transfection of reporter mESC with Cas9-sgRNAs and nCas9-sgRNAs, Cas9^D^ induced a significant percentage of GFP^+^RFP^−^ cells representing STGC and GFP^+^RFP^+^ cells representing LTGC at 4 different target sites as Cas9 (Supplementary Fig. 2b, c). In human U2OS cells, a significant but lower STGC and LTGC were observed (Supplementary Fig. 2d). In both mESC and U2OS cells, nCas9-induced LTGC bias was greater than Cas9-induced LTGC bias (Supplementary Fig. 2c, d). However, the detected LTGC bias in nCas9-induced HR was still far below 100% although HR without a second end for second-end capture or strand annealing would all in theory generate LTGC events. This implies that the second end remains available from a converging replication fork with a significant probability to facilitate termination of nCas9-induced HR by STGC, thus suppressing LTGC.

To further determine the effect of the *GFP* homology in the second end on LTGC suppression, we deleted the *GFP* sequence downstream of the I-SceI site of *I-Sce-GFP* in the SCR-RFP reporter directly in reporter mESC to generate the SCR-dGFP reporter by using paired Cas9-sgRNA approach (Supplementary Fig. 2e, f)^34^. The LTGC bias was increased from 2% with the SCR-RFP reporter to 10% with the SCR-dGFP reporter for Cas9-induced two-ended DSBs and from 10% to 50-70% for nCas9-induced nicks (Supplementary Fig. 2g). This again suggests that not only is the non-invading second end generated but also the homologous *GFP* sequence of this end is critical for the termination in nCas9-induced HR as in HR of two-ended DSBs. We also treated SCR-RFP reporter mESC with Aphidicolin and L-Mimosine. This replication inhibition reduced nCas9-induced STGC to a larger extent than Cas9-induced STGC (Supplementary Fig. 3a). In addition, we initiated bidirectional DNA replication in the SV40 origin-containing SCR-RFP reporter by ectopic expression of SV40 large T antigen (LT) (Supplementary Fig. 3b)^44^. Local DNA replication dramatically increased nCas9-induced HR by over 14 folds, but Cas9-induced HR increased only by 2–3 folds (Supplementary Fig. 3b). These results suggest that HR repair of nCas9-induced nicks is coupled with conversion of nicks into one-ended DSBs by DNA replication.

### *BRCA1* deficiency causes replication strand-asymmetric LTGC bias in repair of one-ended DSBs

To determine whether BRCA1 regulates HR of one-ended DSBs, we induced a site-specific DSB or nick in *Brca1*^*+/+*^ and *Brca1*^*m/m*^ clones (Supplementary Fig. 4a). Consistent with a previous study^18^, I-SceI-induced STGC was impaired in *Brca1*^*m/m*^ clones but the LTGC bias was enhanced by *Brca1* deficiency (Supplementary Fig. 4b). Similarly, Cas9-induced STGC and LTGC at the four different breakage sites were reduced in *Brca1*^*m/m*^ cells (Fig. 2b and Supplementary Fig. 4c). Because the LTGC reduction was smaller than the STGC reduction, Cas9-induced LTGC bias was elevated by about 3–4 folds in cells deficient for *Brca1* (Fig. 2b and Supplementary Fig. 4c). However, while Cas9^D^-induced STGC was reduced in *Brca1*^*m/m*^ cells at all four different nicking sites as compared to *Brca1*^*+/+*^ cells, LTGC was reduced at the gHR1b and gHR2 site, little changed at the gHR3 site and increased at the gHR1a site in *Brca1*^*m/m*^ cells (Fig. 2b and Supplementary Fig. 4c). In contrast, Cas9^H^-induced STGC was reduced at the gHR1b and gHR2 site but little changed at the gHR3 and gHR1a site in *Brca1*^*m/m*^ cells, whereas LTGC was unchanged at the gHR1b site and significantly increased at the other three sites (Fig. 2b and Supplementary Fig. 4c). As a result, in *Brca1*^*m/m*^ cells, the increase of Cas9^H^-induced LTGC bias was much greater than the increase of Cas9^D^-induced LTGC bias (Fig. 2b and Supplementary Fig. 4c). At these 4 targets sites, Cas9^D^ induced nicks on the Crick strand and Cas9^H^ on the Watson strand. Cas9^D^- and Cas9^H^-induced nicks respectively collided with the leading strand and the lagging strand of the rightward fork. As the rightward forks, not the leftward forks, can convert nCas9-induced nicks into one-ended DSBs that generate LTGC-mediated GFP^+^RFP^+^ cells, the stronger effect of *Brca1* deficiency on Cas9^H^-induced LTGC bias suggests that BRCA1 may suppress LTGC bias in a replication strand asymmetry in the repair of one-ended DSBs.

However, because Cas9^D^ and Cas9^H^ induced a nick respectively on the target strand within the DNA-RNA hybrid and on the non-target single strand at the gHR1a, gHR1b, gHR2 and gHR3 target sites, the strand asymmetry in BRCA1-mediated suppression of LTGC bias may not be due to replication, but due to the different conformational contexts of the nCas9-sgRNA-DNA complex. We thus used the EMX1-SCR reporter containing 6 additional nCas9 targeting sites to further test this strand asymmetry (Fig. 2c). In this reporter, a 44-bp human *EMX1* sequence was inserted at 5′ of the I-SceI site of the SCR-RFP reporter by CRISPR/Cas9-induced ssODN-based homology-directed repair^45^, thus generating 6 additional nCas9 targeting sites, where Cas9^D^ or Cas9^H^ can induce a nick colliding with either leading strand or lagging strand of DNA replication. Similar to the four nicking sites tested above, at the sites targeted by gEmx7, gEmx9 and gEmx12, Cas9^D^- and Cas9^H^-induced nicks could be converted into one-ended DSBs by a collision respectively with the leading strand (the lead collapse in Fig. 2d) and with the lagging strand (the lag collapse in Fig. 2d)^29^, leading to HR we could measure. It is opposite for the replication strand colliding with Cas9^D^- and Cas9^H^-induced nicks at the sites targeted by gEmx8, gEmx10, and gEmx11 (Fig. 2d). No matter whether LTGC bias was induced by Cas9^D^ or Cas9^H^, the increase of LTGC bias coupled with the lag collapse was much greater than the increase of LTGC bias with the lead collapse in *Brca1*^*m/m*^ cells as compared to *Brca1*^*+/+*^ cells (Fig. 2e, f and Supplementary Fig. 4c, d). However, no significant difference was detected in BRCA1-mediated suppression of LTGC bias between nicks induced on the target strand in the DNA-RNA hybrid and on the non-target single strand within the nCas9-sgRNA-DNA complex (Supplementary Fig. 5a). This suppression was also little altered between the PAM on the Watson strand and on the Crick strand for nCas9-sgRNAs (Supplementary Fig. 5b). Together, these data suggest that the strand asymmetry in exacerbation of nCas9-induced LTGC bias by *Brca1* deficiency was associated with DNA replication, more strongly with the lagging strand collision than with the leading strand collision, not with the conformational context of the nCas9-sgRNA-DNA complex. As LTGC causes gene amplifications (such as TDs) and SBSs, this bias may lead to asymmetric distribution of these mutations across leading and lagging strands in *BRCA1*-deficient cancers.

### Nicks, not two-ended DSBs, stimulate NHEJ with MH-mediated small deletions in *Brca1*-deficient cells

**Fig. 3:**
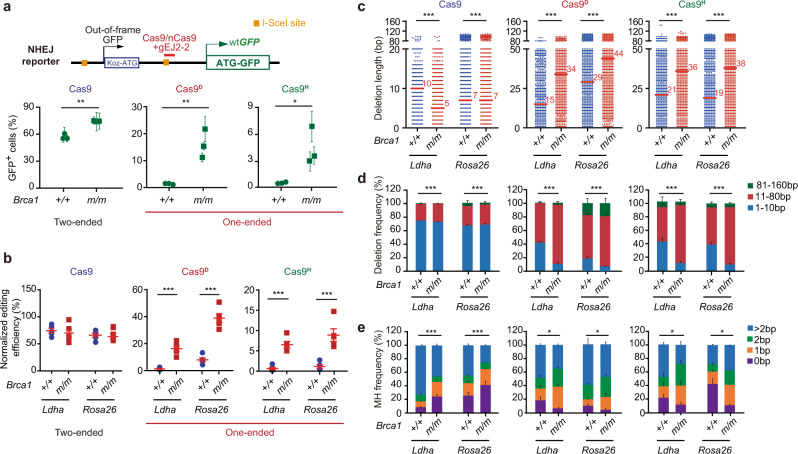
*Brca1* deficiency stimulates nick-induced NHEJ with longer deletions and more MH usage at repair junctions. **a** Cas9- or nCas9-induced NHEJ in multiple isogenic *Brca1*^*+/+*^ and *Brca1*^*m/m*^ mESC clones containing an NHEJ reporter indicated on the top. The NHEJ reporter contains a stronger artificial Kozak-ATG translation start site ‘Koz-ATG’ that causes protein synthesis of out-of-frame GFP. NHEJ of Cas9- or nCas9-induced breaks in the reporter could generate GFP^+^ cells by GFP reframing. Solid green circles and squares indicate independent clones. **b** Cas9- or nCas9-induced NHEJ at intron 5 of mouse *Ldha* and intron 2 of *Rosa26* locus in *Brca1*^*+/+*^ and *Brca1*^*m/m*^ mESC. The NHEJ-mediated editing efficiency is calculated as ratios of edited reads to total reads from targeted Illumina sequencing and normalized by respective transfection efficiencies. Solid circles and squares indicate independent experiments (*n* = 5). **c**–**e** Analysis of NHEJ junctions for deletion length with the median length indicated (**c**), distribution of deletions with three size ranges (**d**) and the usage of MH (**e**) between *Brca1*^*+/+*^ and *Brca1*^*m/m*^ mESC. Statistics is performed by One-way ANOVA with Tukey’s multiple comparison test for (**a**, **b**). Columns indicate the mean ± S.E.M from five independent experiments, and statistical significance is detected by two-tailed Mann–Whitney test for (**c**–**e**). ^*^P < 0.05; ^**^P < 0.01 and ^***^P < 0.001.

In *Brca1*-deficient mESC, the delayed availability of the second ends would facilitate NHEJ due to inefficient HR (Supplementary Fig. 6a). We thus used mESC harboring a single-copy NHEJ reporter previously established to test this possibility^46^. In this NHEJ reporter containing a *GFP* expression cassette driven by the *phosphoglycerate kinase* (*PGK*) promoter (Supplementary Fig. 6b), no in-frame GFP is translated due to an upstream, out-of-frame translation start site Kozak-ATG (‘Koz-ATG’) flanked by two sequentially positioned I-SceI sites^47^. A site-specific DSB can be induced between “Koz-ATG” and the *ATG-GFP* coding region by Cas9, allowing repair by mutagenic NHEJ to generate indels at the repair junction^46^. Generally, indels with a net addition of “3n + 2” bp or net loss of “3n-1” bp could change the 34-bp frame-shift to in-frame, leading to the production of GFP^+^ cells. Direct indels of any nucleotide within the ATG of Koz-ATG could also allow normal translation of ATG-GFP, making cells GFP^+^. The frequency of GFP^+^ cells therefore represents the NHEJ efficiency in cells. NHEJ repair of two-ended DSBs induced by Cas9 was highly efficient at 4 target sites by gEJ2-2, gEJ2a, gEJ2-3, and gEJ2-4, whereas the level of Cas9^D^- or Cas9^H^-induced NHEJ was much smaller but detectable (Supplementary Fig. 6c). We then generated isogenic *Brca1*^*+/+*^ and *Brca1*^*m/m*^ NHEJ reporter mESC clones (Supplementary Fig. 6d). NHEJ of two-ended DSBs at the gEJ2-2 target site was slightly increased in *Brca1*^*m/m*^ clones (Fig. 3a). However, the frequencies of Cas9^D^- and Cas9^H^-induced NHEJ at the same site were greatly elevated by more than 10-fold when *Brca1* was deficient (Fig. 3a), indicating that NHEJ serves as an important alternative pathway in repair of one-ended DSBs when BRCA1-mediated HR is defective.

We also induced a site-specific DSB or nick at the natural *Ldha* and *Rosa26* loci and examined the frequency and junctions of NHEJ by deep sequencing. The frequency of Cas9-induced NHEJ was not affected by *Brca1* status, but nCas9-induced NHEJ was strongly stimulated by *Brca1* deficiency (Fig. 3b). The deletion lengths in Cas9-induced NHEJ were shorter in *Brca1*^*m/m*^ cells than in *Brca1*^*+/+*^ cells (Fig. 3c). The proportions of <11 bp deletions in *Brca1*^*m/m*^ cells were similar to *Brca1*^*+/+*^ cells (Fig. 3d). The use of MH was reduced in *Brca1*^*m/m*^ cells (Fig. 3e). These data were consistent with the function of BRCA1 in end resection. In contrast, in nCas9-induced NHEJ, the median deletion lengths were larger, and <11 bp deletions were much less in *Brca1*^*m/m*^ cells than in *Brca1*^*+/+*^ cells (Fig. 3c, d). However, *Brca1* deficiency increased the proportion of 11-80 bp deletions and the MH usage (Fig. 3d, e).

Similarly, using SCR-RFP reporter U2OS cells, we also analyzed whether *BRCA1* deficiency reduced HR and stimulated nCas9-induced NHEJ in human cells. As expected, siRNA-mediated depletion of *BRCA1* reduced Cas9- and Cas9^D^-induced HR in U2OS cells (Supplementary Fig. 7a, b). Similar to *Brca1*^*m/m*^ mESC as compared to *Brca1*^*+/+*^ mESC, Cas9^D^-induced NHEJ at the loci of *AAVS1* and *EMX1*, not Cas9-induced NHEJ, was increased by *BRCA1* depletion (Supplementary Fig. 7c). *BRCA1* siRNA had little effect on the deletion lengths and the proportion of 11–80 bp deletions in Cas9-induced NHEJ, but reduced the MH use at the junctions (Supplementary Fig. 7d–f). In contrast, the median deletion lengths, the proportions of 11–80 bp deletions and the MH use at the junctions were increased in Cas9^D^-induced NHEJ in *BRCA1*-depleted U2OS cells (Supplementary Fig. 7d–f). Together, these results indicate that nCas9-induced small deletions in both mouse and human cells deficient for *BRCA1*, not Cas9-induced small deletions, resemble those of spontaneous small deletions enriched in *BRCA1*-deficient tumors.

### Nicks, not two-ended DSBs, promote translocations associated with *BRCA1* deficiency

**Fig. 4:**
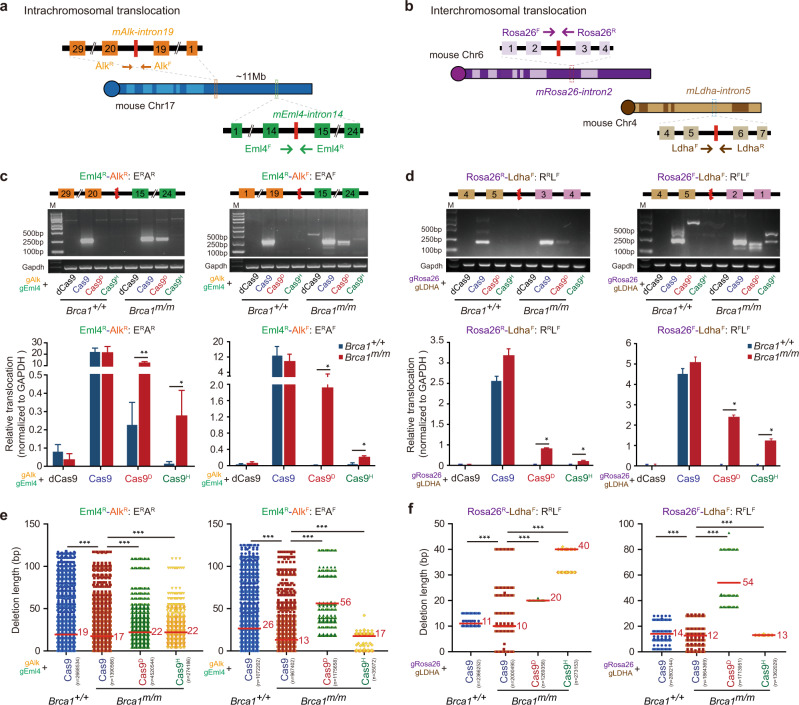
BRCA1 suppresses nick-induced translocation. **a**, **b** Schematic for induction of intrachromosomal translocations between the *Alk* and *Eml4* loci (**a**) or interchromosomal translocations between the *Rosa26* and *Ldha* loci (**b**) in mESC. Red lines indicate the Cas9 or nCas9 target sites flanked by PCR primers denoted in an arrowed line for translocation detections. **c**, **d** PCR products and quantification of *Eml4–Alk* translocations (**c**) and *Rosa26–Ldha* translocations (**d**) in *Brca1*^*+/+*^ and *Brca1*^*m/m*^ mESC transfected with dCas9, Cas9 or nCas9. Schematics of *Eml4–Alk* translocations E^R^A^R^ and E^R^A^F^ (**c**) and *Rosa26–Ldha* translocations R^R^L^F^ and R^F^L^F^ (**d**) are shown on top. PCR products of translocations and *Gapdh* as an internal PCR control (middle) were mixed in the ratio of 10:1 in volume for Illumina sequencing. Translocations were quantified as relative translocation levels, which are ratios of translocation reads to *Gapdh* reads and normalized by transfection efficiencies (bottom). The fold of increase between *Brca1*^*+/+*^ and *Brca1*^*m/m*^ cells is indicated above each column. **e**, **f** Deletion length at the junctions of *Eml4–Alk* translocations E^R^A^R^ and E^R^A^F^ (**e**) and *Rosa26–Ldha* translocations R^R^L^F^ and R^F^L^F^ (**f**) between *Brca1*^*+/+*^ and *Brca1*^*m/m*^ mESC. The median length is indicated. Total reads (*n*) for translocations are indicated under Cas9 or nCas9 in parenthesis. Each dot represents 500 reads. Columns indicate the mean ± S.E.M from three independent experiments. Statistical significance is detected by two-tailed Student’s *t* test. To compare the deletion distribution between each sample, two-tailed Mann–Whitney test is performed. ^*^P < 0.05; ^**^P < 0.01 and ^***^P < 0.001.

As translocations are accumulated as an SV signature in *Brca1*-deficient cancers^2,3^, we induced intrachromosomal and interchromosomal translocations in both *Brca1*^*+/+*^ and *Brca1*^*m/m*^ mESC. Concomitant DSBs or nicks, one at intron 14 of *Eml4* and the other at intron 19 of *Alk*, were generated in mESC respectively by Cas9 or nCas9 to induce four types of intrachromosomal translocations: Eml4^F^-Alk^F^ (E^F^A^F^), Eml4^F^-Alk^R^ (E^F^A^R^), Eml4^R^-Alk^F^ (E^R^A^F^) and Eml4^R^-Alk^R^ (E^R^A^R^) (Fig. 4a). Similarly, four types of interchromosomal translocations Rosa26^F^-Ldha^F^ (R^F^L^F^), Rosa26^F^-Ldha^R^ (R^F^L^R^), Rosa26^R^-Ldha^F^ (R^R^L^F^) and Rosa26^R^-Ldha^R^ (R^R^L^R^) were induced between intron 2 of *Rosa26* and the other at intron 5 of *Ldha* (Fig. 4b). Translocations were detected by PCR and quantified by deep sequencing (Fig. 4c, d). In *Brca1*^*+/+*^ cells, Cas9-induced translocations were efficient whereas nCas9-induced translocations were hardly detected (Fig. 4c, d). However, in *Brca1*^*m/m*^ cells, while Cas9-induced translocations including E^R^A^R^, E^R^A^F^, R^R^L^F^ and R^F^L^F^ were induced as efficiently as in *Brca1*^*+/+*^ cells, nCas9-induced translocations of the same types were elevated by 30–60,000 times in *Brca1*^*m/m*^ cells (Fig. 4c, d). Together, these results indicate *Brca1* deficiency has little effect on the generation of translocations induced by two-ended DSBs, but greatly stimulates translocations induced by DNA nicks.

Junction analysis of Cas9-induced translocations revealed that nearly all translocation events in *Brca1*^*+/+*^ mESC were generated with indels (Supplementary Fig. 8a, b). However, in *Brca1*^*m/m*^ cells, an increased fraction of translocations was generated without indels (Supplementary Fig. 8a, b). The deletions at the Cas9-induced junctions were smaller and the frequency of MH used was greatly lower in *Brca1*^*m/m*^ cells (Fig. 4e, f and Supplementary Fig. 8c, d). These results suggest that BRCA1 does not determine the frequency of Cas9-induced translocations but instead promotes end resection and the use of MH.

Unlike Cas9-induced translocations, nCas9-induced translocations in *Brca1*^*m/m*^ cells engaged little precise end joining (Supplementary Fig. 8a, b). The deletions at the junctions were different from those in Cas9-induced translocations and the deletion length in most cases was longer (Fig. 4e, f). Also, in *Brca1*^*m/m*^ cells, the MH usage in each type of nCas9-induced translocations was generally more frequent than in Cas9-induced translocations (Supplementary Fig. 8c, d). Therefore, these data indicate that translocations induced by DNA nicks had more extensive end resection and more frequent use of MH than translocations induced by two-ended DSBs in *Brca1*-deficient cells.

### BRCA1 suppresses TDs mediated by two-round homologous strand invasion

**Fig. 5:**
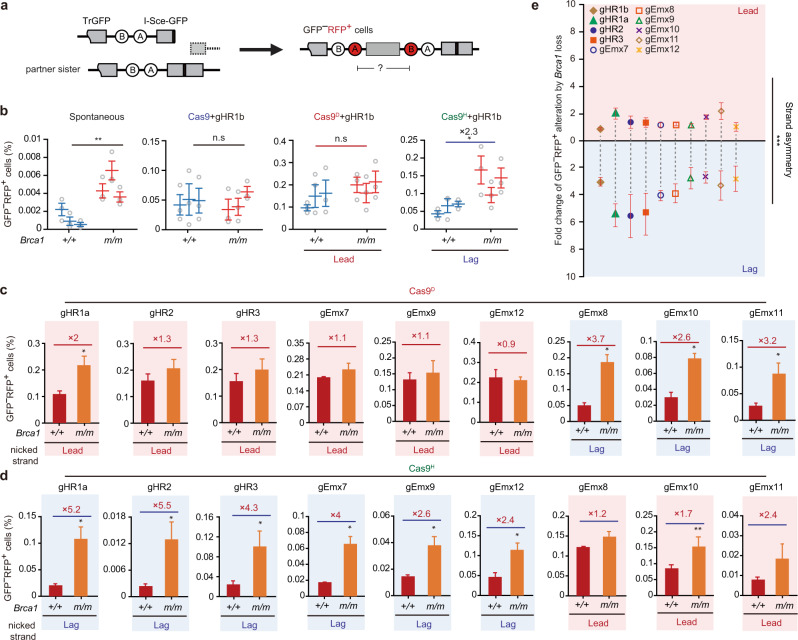
BRCA1 suppresses nick-induced TDs in a strand-biased manner. **a** Schematic of GFP^−^RFP^+^ products generated from the SCR-RFP reporter. **b** Spontaneous, Cas9-gHR1b- and nCas9-gHR1b-induced GFP^−^RFP^+^ products in three of *Brca1*^*+/+*^ and *Brca1*^*m/m*^ clones containing the SCR-RFP reporter. **c**, **d** Frequency of GFP^−^RFP^+^ cells induced by Cas9^D^ (**c**) or Cas9^H^ (**d**) together with one of 9 sgRNAs in *Brca1*^*+/+*^ and *Brca1*^*m/m*^ cells. The replication strand that encounters nCas9-induced nicks is indicated. The fold of increase between *Brca1*^*+/+*^ and *Brca1*^*m/m*^ cells is stated above each pair of columns for indicated nCas9-sgRNA. **e** Combined analysis of strand asymmetry in altering the frequency of nick-induced GFP^−^RFP^+^ cells by *Brca1* deficiency. The replication strand that encounters nCas9-induced nicks is indicated. Each dot indicates one independent experiment and statistics is performed by One-way ANOVA with Tukey’s multiple comparison test in (**b**). Columns indicate the mean ± S.E.M from three independent experiments and statistics is performed by two-tailed Student’s *t* testing (**c**, **d**). Each symbol represents the mean of at least three independent experiments for one sgRNA and statistics is performed by two-tailed Student’s *t* testing (**e**). ^*^P < 0.05; ^**^P < 0.01 and ^***^P < 0.001.

In the SCR-RFP reporter, site-specific stalling of replication forks induced GFP^−^RFP^+^ cells, which represent excess ~10 kb MH-mediated TDs^24^. However, both spontaneous and I-SceI-induced GFP^−^RFP^+^ cells are products from *GFP* repeat-mediated TD, containing three copies of *GFP* with a ~10-kb duplication span^48,49^. To determine which type of TDs represented by GFP^−^RFP^+^ cells is induced by DNA nicks and could be promoted by *BRCA1* deficiency, we first analyzed the frequencies of GFP^−^RFP^+^ cells induced spontaneously or by nCas9 and Cas9 (Fig. 5a). Spontaneous GFP^−^RFP^+^ cells were scarce but increased by ~4-fold in *Brca1*^*m/m*^ clones (Fig. 5b). Expression of Cas9-gHR1b generated a higher level of GFP^−^RFP^+^ cells, which was unaffected by *Brca1* mutation as induction of GFP^−^RFP^+^ cells by I-SceI (Fig. 5b)^24^. In contrast, the frequencies of GFP^−^RFP^+^ cells induced by Cas9^H^, not those by Cas9^D^, were stimulated by *Brca1* deficiency (Fig. 5b), indicating a strand asymmetry.

We then used the “EMX1-SCR” reporter to further determine the association of the strand asymmetry with DNA replication (Supplementary Fig. 9a). *Brca1* deficiency caused varying effects on Cas9-induced GFP^−^RFP^+^ cells (Supplementary Fig. 9b, c). It stimulated GFP^−^RFP^+^ cells induced by Cas9^H^, not those by Cas9^D^, at the gHR1a, gHR2, gHR3, gEmx7, gEmx9 and gEmx12 sites, where nicks induced by Cas9^H^ (not by Cas9^D^) caused the lag collapse and generated GFP^−^RFP^+^ cells (Fig. 5c, d). However, at the gEmx8, gEmx10 and gEmx11 sites, Cas9^D^-induced GFP^−^RFP^+^ cells were stimulated more than Cas9^H^-induced GFP^−^RFP^+^ cells by *Brca1* deficiency (Fig. 5c, d). Cas9^D^- and Cas9^H^-induced GFP^−^RFP^+^ cells at these three target sites arose respectively from the lag and the lead collapse of DNA nicks. Thus, the combined data from these 10 sites indicated that BRCA1-mediated suppression of nCas9-induced GFP^−^RFP^+^ cells was biased towards the lagging strand collision, not the leading strand collision (Fig. 5e), suggesting a replication strand asymmetry in this BRCA1 function. Such asymmetry was further validated by excluding the potential effects that the conformational context of the nicked strand in the nCas9–sgRNA–DNA complex or the PAM position for Cas9 might have (Supplementary Fig. 9d, e).

**Fig. 6:**
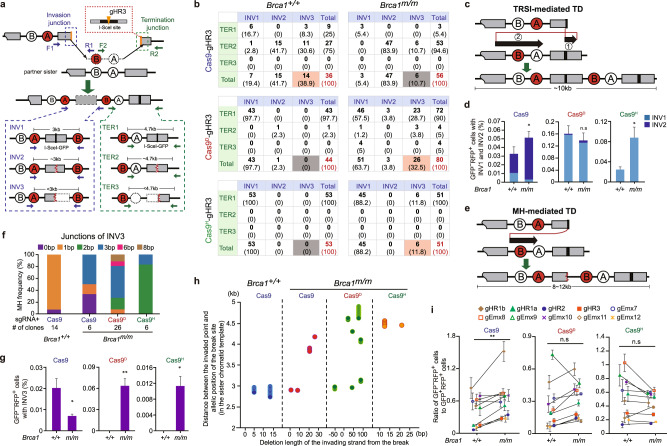
Nick-induced TDs suppressed by BRCA1 are mediated either by two-round strand invasion or by MH. **a** Structural classification of TD products induced by Cas9-gHR3 and nCas9-gHR3. After induction of a two-ended or one-ended DSB by Cas9-gHR3 and nCas9-gHR3, results in TDs (i.e., GFP^−^RFP^+^ cells), three types of invasion junctions, i.e., INV1, INV2 and INV3 (blue dotted frame), can be determined and classified by PCR with primers F1 and R1 as indicated by blue arrows and three types of termination junctions, i.e., TER1, TER2 and TER3 (green dotted frame), with primers F2 and R2 as indicated by green arrows. The straight black line and curve red lines within *I-Sce-GFP* indicate an intact and mutated I-SceI site, respectively. The dotted square and circle in INV3 and TER3 denote deletion. **b** Number (in bold) of Cas9- or nCas9-induced TD events with each combined type of invasion and termination junctions in *Brca1*^*+/+*^ and *Brca1*^*m/m*^ cells. The portion of each combined type in GFP^−^RFP^+^ cells analyzed is shown in parenthesis. **c** TRSI-mediated TD model. The first homologous strand invasion into allelic *I-Sce-GFP* restores the I-SceI site and the second homologous strand invasion into *TrGFP* allows TD of the *RFP* exon B and A cassette. **d** Frequency of Cas9- and nCas9-induced TRSI-mediated TDs in *Brca1*^*+/+*^ and *Brca1*^*m/m*^ cells. TRSI-mediated TDs are characterized by GFP^−^RFP^+^ cells with INV1 or INV2. The frequency is calculated as the overall frequency of GFP^−^RFP^+^ cells × the proportion of INV1 or INV2 in GFP^−^RFP^+^ cells analyzed. **e** MH-mediated TD model. MH-mediated invasion that occurs upstream of exon B allows TD of the *RFP* exon B and A cassette but generates a nested *GFP* that contains only the first half of *I-Sce-GFP*. **f** MH distribution at invasion junctions of Cas9- and nCas9-induced MH-mediated TDs in *Brca1*^*+/+*^ and *Brca1*^*m/m*^ cells. MH-mediated TDs are characterized by GFP^−^RFP^+^ cells with INV3. The number for MH-mediated TD events analyzed is indicated under each column. **g** Frequency of Cas9- and nCas9-induced MH-mediated TDs in *Brca1*^*+/+*^ and *Brca1*^*m/m*^ cells. The frequency is calculated as the overall frequency of GFP^−^RFP^+^ cells × the proportion of INV3 in GFP^−^RFP^+^ cells analyzed. **h** The processed length of Cas9- and nCas9-induced breaks (x-axis) and distance of MH-mediated invasion point to allelic position of Cas9- and nCas9-induced breaks in sister chromatid (*y*-axis) in MH-mediated TDs in *Brca1*^*+/+*^ and *Brca1*^*m/m*^ cells. One circle denotes one TD event. **i** Effect of *Brca1* deficiency on ratios of GFP^−^RFP^+^ cells to GFP^+^RFP^+^ cells induced by Cas9 and nCas9 at each of 10 independent sites as indicated. Columns indicate the mean ± S.E.M from three independent experiments and statistics is performed by two-tailed Student’s *t* testing (**d**, **h**). Each symbol represents the ratio at one site and statistics is performed by two-tailed Student’s *t* test in **i**. ns, *P* > 0.05; ^*^P < 0.05; ^**^P < 0.01.

In the GFP^−^RFP^+^ products, the second (“nested”) and third *GFP* copies reflect the steps of invasion and termination in repair of the damaged *I-Sce-GFP*, respectively (Fig. 6a). To determine the TD types that lead to GFP^−^RFP^+^ cells, we further analyzed the sequences of these two *GFP* copies in GFP^−^RFP^+^ products and identified three classes of invasion and termination in these GFP^−^RFP^+^ products with respect to GFP alterations: Invasion Class 1 (INV1) or Termination Class I (TER1) for the *I-Sce-GFP* sequence with no indels at the break point, INV2 or TER2 for the *I-Sce-GFP* sequence with indels at the break point, INV3 for the nested *GFP* with only the first half of *I-Sce-GFP* sequence from the break point and TER3 for the third *GFP* with only the second half of *I-Sce-GFP* from the break point (Fig. 6a).

In all 41 spontaneous GFP^−^RFP^+^ products from *Brca1*^*m/m*^ mESC, the nested *GFP* and the third *GFP* are respectively INV1 and TER1, i.e. *I-Sce-GFP* with intact I-SceI site (Supplementary Fig. 10a). Two different mechanisms, i.e., typical non-allelic SCR and two-round strand invasion (TRSI) model, could mediate spontaneous generation of these GFP^−^RFP^+^ products (Supplementary Fig. 10b). Due to the longer 3’ side of the I-SceI site, spontaneous DSBs could occur with a higher probability downstream of the I-SceI site of *I-Sce-GFP* to induce more spontaneous GFP^−^RFP^+^ cells than spontaneous GFP^+^RFP^+^ cells via non-allelic SCR/LTGC (Supplementary Fig. 10c)^49^.

Because Cas9-induced DSBs around the I-SceI site, it was unlikely for non-allelic SCR/LTGC to generate GFP^−^RFP^+^ cells. Indeed, Cas9-induced GFP^−^RFP^+^ cells were mainly INV1 or INV2 products (Fig. 6b). This is consistent with TRSI-mediated TD (Fig. 6c). In *Brca1*^*+/+*^ mESC, Cas9-induced GFP^−^RFP^+^ products included 19.4% INV1, 41.7% INV2/TER2 and 38.9% INV3 (Fig. 6b). In *Brca1*^*m/m*^ mESC, over 80% of Cas9-induced GFP^−^RFP^+^ products were INV2/TER2, with much smaller proportions of INV1/TER1 and INV3/TER3 products (Fig. 6b). Surprisingly, both the nested *GFP* and the third *GFP* shared the same indels in 41 out of 47 INV2/TER2 products from *Brca1*^*m/m*^ mESC (Supplementary Data 1). Two identical mutations could occur independently but rarely on both *I-Sce-GFP* of INV1/TER1 products to generate the INV2/TER2 products in TRSI-mediated TDs (Model 1 in Supplementary Fig. 10d). Thus, the *I-Sce-GFP* copy in the sister chromatid template could first be cleaved by Cas9 and mutated by NHEJ prior to first-round invasion. The mutation generated could then be introduced into the nested and the third *GFP* in INV2/TER2 products by TRSI (Model 2 in Supplementary Fig. 10d).

In addition, 43 of 44 Cas9^D^-induced GFP^−^RFP^+^ products and all of 53 Cas9^H^-induced GFP^−^RFP^+^ products were INV1/TER1 in *Brca1*^*+/+*^ mESC (Fig. 6b). In comparison, *Brca1*^*m/m*^ mESC had a smaller portion of INV1 in Cas9^D^- and Cas9^H^-induced GFP^−^RFP^+^ products (Fig. 6b). INV2 rarely occurred in nCas9-induced GFP^−^RFP^+^ products in either *Brca1*^*+/+*^ or *Brca1*^*m/m*^ mESC and even no INV2 were detected in Cas9^H^-induced GFP^−^RFP^+^ products (Fig. 6b). The rare occurrence of INV2 in nCas9-induced GFP^−^RFP^+^ cells was consistent with a lower rate of nCas9-induced targeted mutations prior to first-round strand invasion. In addition, the combined frequency of Cas9-induced INV1 and INV2 was higher in *Brca1*^*m/m*^ mESC than in *Brca1*^*+/+*^ mESC (Fig. 6d), suggesting BRCA1 suppress TRSI-mediated TD in the repair of two-ended DSBs. While the combined frequency of Cas9^D^-induced INV1 and INV2 was marginally smaller in *Brca1*^*m/m*^ mESC than in *Brca1*^*+/+*^ mESC, Cas9^H^ induced a higher frequency of INV1 in *Brca1*^*m/m*^ mESC than in *Brca1*^*+/+*^ mESC (Fig. 6d). This suggests a strong strand asymmetry in BRCA1-mediated suppression of nick-induced TRSI-mediated TD.

### Nicks, not two-ended DSBs, induce increased MH-mediated TDs associated with *BRCA1* deficiency

A small fraction of induced GFP^−^RFP^+^ cells were classified as an INV3 product, in which the left end of DSBs on *I-Sce-GFP* could use the MH upstream of the exon B in sister chromatid to mediate extensive DNA synthesis, thus creating the nested *GFP* that contains only the first half of *I-Sce-GFP* (Fig. 6e). While TER1 and TER2 are resolved by the termination step of HR (Supplementary Fig. 10d), the extended 3’ end of nascent DNA could be prematurely displaced from the template of DNA synthesis upstream of *I-Sce-GFP* and joined with the second end generating TER3 (Fig. 6a and Supplementary Fig. 10e). In each combination of INV3 with TER1, TER2 or TER3, MH-mediated TDs span ~8–12 kb (Fig. 6e). MH analysis at the entry site for the invading ends revealed that MH is used in 13 out of 14 Cas9-induced INV3 products in *Brca1*^*+/+*^ cells, 4 out of 6 in Cas9-induced INV3 products and all 32 nCas9-induced INV3 products in *Brca1*^*m/m*^ cells (Fig. 6b, f). In *Brca1*^*+/+*^ cells, the sizes of MH varied from 1 bp to 8 bp, but smaller in average in Cas9-induced INV3 than in nCas9-induced INV3 (Fig. 6f). These results indicate these nCas9-induced TDs are MH-mediated, resembling those associated with *BRCA1* deficiency, but differ from Cas9-induced INV3.

In *Brca1*^*+/+*^ cells, 14 out of 36 of Cas9-induced GFP^−^RFP^+^ products were INV3, but none for nCas9-induced GFP^−^RFP^+^ products (Fig. 6b), implying MH-mediated TD in the repair of one-ended DSBs is significantly suppressed in normal cells. More importantly, among Cas9-induced GFP^−^RFP^+^ cells, the INV3 products were proportionally more in *Brca1*^*+/+*^ cells than in *Brca1*^*m/m*^ cells (Fig. 6b). The absolute frequency of Cas9-induced INV3 was reduced by 3-fold in *Brca1*^*m/m*^ mESC (Fig. 6g), demonstrating that two-ended DSBs may not be a source for induction of MH-mediated TDs associated with *BRCA1* mutations. Compared with no INV3 in *Brca1*^*+/+*^ mESC, Cas9^D^ and Cas9^H^ generated a significant level of INV3 among the GFP^−^RFP^+^ products in *Brca1*^*m/m*^ cells (Fig. 6b). The absolute frequency of Cas9^D^- and Cas9^H^-induced INV3 products was also significant in *Brca1*^*m/m*^ cells, respectively at 0.066% and 0.012% (Fig. 6g), indicating BRCA1-mediated suppression of nick-induced INV3 formation. Together, these results suggest that DNA nicks, not two-ended DSBs, induce MH-mediated TDs associated with *BRCA1* deficiency. In Cas9-induced MH-mediated TDs, *Brca1* deficiency caused a greater loss of nucleotides in the invading ends and shifted the entry site for MH-mediated strand invasion further away from the allelic position of the break site (Fig. 6h). This pattern of alterations was retained in nCas9-induced MH-mediated TDs in *Brca1*^*m/m*^ cells (Fig. 6h).

In the SCR-RFP reporter, BRCA1 may have a preference in suppressing nCas9-induced TDs mediated by either non-allelic SCR, TRSI- or MH-mediated mechanism (Supplementary Fig. 11a). Among Cas9^D^- and Cas9^H^-induced GFP^−^RFP^+^ cells, the proportions of INV3, a product of MH-mediated TD, were increased from none in both in *Brca1*^*+/+*^ cells to 32.5% and 11.8% in *Brca1*^*m/m*^ cells, respectively, whereas *Brca1* deficiency stimulated nCas9-induced INV1 and INV2, a product of TRSI-mediated TD, to a lesser extent (i.e., Cas9^H^-induced INV1) or not at all (i.e., Cas9^H^-induced INV1 and INV2) (Fig. 6b). This indicates BRCA1 control the balance between TRSI- and MH-mediated TD with a suppressive bias towards MH-mediated TD in the repair of nick-induced DSBs. In *Brca1*^*+/+*^ mESC, Cas9 or nCas9 generally generated fewer GFP^−^RFP^+^ cells than GFP^+^RFP^+^ cells (Fig. 6i and Supplementary Fig. 11b). This suggests that non-allelic SCR/LTGC is more likely used over TRSI- and MH-mediated TD to repair Cas9- and nCas9-induced breaks. We also compared the ratios of GFP^−^RFP^+^ cells to GFP^+^RFP^+^ cells between *Brca1*^*+/+*^ and *Brca1*^*m/m*^ mESC. *Brca1* deficiency elevated the ratios of GFP^−^RFP^+^ cells to GFP^+^RFP^+^ cells induced by Cas9 at 8 of 10 target sites (Fig. 6i), suggesting that BRCA1 promotes the choice of non-allelic SCR/LTGC over TRSI- and MH-mediated TD in repair of Cas9-induced DSBs. However, the ratios of GFP^−^RFP^+^ cells to GFP^+^RFP^+^ cells induced by Cas9^D^ or Cas9^H^ were not elevated by *Brca1* deficiency (Fig. 6i). This indicates a difference in BRCA1-mediated control of pathway choices between repair of nCas9-induced nicks and Cas9-induced two-ended DSBs.

## Discussion

Defective HR repair of two-ended DSBs due to *BRCA1* mutation is often regarded as a cause of characteristic mutational signatures in *BRCA1*-deficient tumors. However, by comparing the repair outcomes for directly two-ended DSBs induced by Cas9 between *Brca1*^*+/+*^ and *Brca1*^*m/m*^ cells, this study demonstrated that this type of DSBs does not induce increased accumulation of mutational signatures resembling those in *BRCA1*-deficient tumors. Instead, as one of the most frequent endogenous DNA lesions in mammalian cells, DNA nicks could be converted into one-ended DSBs by DNA replication and become a major inducer of mutational signatures associated with *BRCA1* deficiency. By exploiting the long duration of Cas9-sgRNA post-cleavage target residence^45,50,51^, together with fast doubling time and short G1 phase of mESC, we increased the probability of a collision between nCas9-induced nicks and DNA replication forks, readily generating one-ended DSBs. Due to *BRCA1* mutation, one-ended DSBs that are supposed to be efficiently repaired by BRCA1-mediated SCR could be left unrepaired, increasing the opportunity for alternative repair pathways (Supplementary Fig. 12)^9,13^. In addition to providing an approach to study repair of nick-converted one-ended DSBs in a site-specific manner, we found alternative repair of these DSBs accumulates mutational signatures resembling those in *BRCA1*-deficient tumors.

First, nCas9-induced NHEJ was stimulated by up to 20 folds in *BRCA1*-deficient cells, indicating that one-ended DSBs could be repaired by NHEJ when HR is defective. The junctions of such NHEJ had more end processing in *BRCA1*-deficient cells, suggesting that this alternative repair pathway does not require BRCA1-mediated end resection. The deletion length and MH usage of these NHEJ products exhibit a pattern similar to the ID6 signature in *BRCA1*-deficient tumors^5,7^. The ID6 signature may be a direct result of MH-mediated end joining (MMEJ), particularly TMEJ^8–12^. The frequent use of MH in NHEJ repair of replication-coupled nick-converted DSBs in *Brca1*-deficient cells suggests that MMEJ or TMEJ may be actively involved; but this possibility along with the underlying mechanism is yet to be confirmed. In contrast, *BRCA1*-deficient cells do not preferentially accumulate Cas9-induced NHEJ products, which also show an indel pattern distinct from the ID6 signature. Second, our data demonstrated that translocations between two distant nicks, not those between two distant two-ended DSBs, were stimulated by *BRCA1* deficiency. This indicates that some unrepaired one-ended DSBs accumulated in *BRCA1*-deficient cells could be repaired by MH-mediated translocations. While two-ended DSBs could lead to reciprocal translocation, this is unlikely for nick-induced one-ended DSBs owing to the lack of two simultaneous ends. Consistent with the role of *BRCA1* in fork protection, not in end resection, the junctions of translocations in *BRCA1*-deficient cells exhibited more end processing in the repair of one-ended DSBs. Thirdly, analysis of spontaneous, Cas9- and nCas9-induced GFP^−^RFP^+^ cells revealed that only a small fraction of Cas9- and nCas9-induced GFP^−^RFP^+^ cells resemble MH-mediated ~10 kb TDs associated with *BRCA1* deficiency. nCas9-induced MH-mediated TDs were greatly stimulated in *BRCA1*-deficient cells whereas Cas9-induced MH-mediated TDs were instead inhibited, suggesting that DNA nicks, not two-ended DSBs, induce *BRCA1*-linked TDs.

Unlike classic DNA nicks that are readily exposed, nCas9-induced nicks are buried within the nCas9-sgRNA-DNA ternary complex^45,50,51^. It is unclear whether cells could sense this type of nicks before nCas9-sgRNA is dissociated spontaneously or by forces from nicked DNA targets. However, long residence of nCas9-sgRNA at nicked targets increases the probability of a collision between nicks and DNA replication forks, promoting the occurrence of replication-coupled one-ended DSBs. Given the different contexts of the nCas9-sgRNA-DNA complex, a nick could be induced on the target strand in the DNA-RNA hybrid by Cas9^D^ or on the non-target single strand by Cas9^H^. In addition, the nCas9-sgRNA complex could use different sides of the structure to engage the collision with a replication fork due to the PAM position for nCas9, either on the Watson strand or on the Crick strand, as does in the collision of dCas9 with transcription^37,52^. Combined with two different colliding strands, i.e., the leading strand and the lagging strand, the conversion of nCas9-induced nicks into one-ended DSBs varies significantly between different contexts. It is possible that the repair of one-ended DSBs could be differently regulated because of the conversion contexts and warrant further investigation in this regard. Nevertheless, in this study, by analyzing the effects of different conversion contexts on BRCA1-mediated suppression of LTGC bias, we found that nCas9-induced LTGC bias was suppressed by BRCA1 in a replication strand asymmetry, more strongly with the lagging strand collision than with the leading strand collision, not with the conformational context of the nCas9-sgRNA-DNA complex. A similar strand asymmetry was also identified in BRCA1-mediated suppression in the generation of nCas9-induced GFP^−^RFP^+^ cells, which represent products by TRSI-mediated TD and MH-mediated TD.

The human genome contains many directly oriented repeats. These repeats can potentially lead to DNA rearrangements such as deletions between repeats, TDs of intervening segments and repeat triplications, destabilizing the genome and causing human diseases^19^. In this study, we used the SCR-RFP reporter to analyze repeat-directed TDs of the intervening *RFP* exon B and A cassette induced by DNA nicks and found that this type of TDs can be further distinguished by the underlying “LTGC” mechanisms including typical non-allelic SCR/LTGC and TRSI-mediated TDs (Supplementary Fig. 11a). Cas9- and nCas9-induced GFP^+^RFP^+^ cells are known to be products of repeat-directed TD by typical non-allelic SCR. In contrast, while allelic homology is expected to be the most preferred template for HR, the invading ends could be prematurely dissociated from homologous templates after allelic strand invasion followed by limited DNA synthesis in yeast^53,54^. The displaced strands that restore the damaged site could reinvade into non-allelic *GFP* in the sister chromatid template, allowing us to capture and detect the preceding allelic strand invasion in LTGC-mediated TD products (i.e., GFP^−^RFP^+^ cells). Thus, after detecting the INV1 and INV2 structures in the majority of Cas9- and nCas9-induced GFP^−^RFP^+^ cells in *Brca1*^*+/+*^ mESC, we proposed the TRSI mechanism for the generation of these GFP^−^RFP^+^ TDs. In addition, only a minority of Cas9- and nCas9-induced GFP^−^RFP^+^ cells are products of MH-mediated TD, and even none of nCas9-induced GFP^−^RFP^+^ cells are generated in *Brca1*^*+/+*^ cells. This suggests that MH-mediated TD in *Brca1*^*+/+*^ mESC is strongly suppressed in the repair of nCas9-induced DSBs but not Cas9-induced DSBs. Nevertheless, these repair choices dictated by strand invasions are applicable to DSB repair in the human genome, particularly in the presence of directly oriented repeats, leading to different types of TDs and triplications^17^.

Does BRCA1 function in these choices? In Cas9-induced HR, *Brca1* deficiency not only elevated the ratios of GFP^−^RFP^+^ cells to GFP^+^RFP^+^ cells but also lowered the proportion of MH-mediated TDs among GFP^−^RFP^+^ cells. In contrast, *Brca1* deficiency hardly affected the ratios of GFP^−^RFP^+^ cells to GFP^+^RFP^+^ cells but increased the proportion of MH-mediated TDs among GFP^−^RFP^+^ cells from none to a detectable level in nCas9-induced HR. This suggests that BRCA1 suppresses MH-mediated TD proportionally the most among non-allelic SCR/LTGC, TRSI- and MH-mediated TD in HR repair of nCas9-induced DSBs.

Our data also revealed a replication strand asymmetry in BRCA1-mediated suppression of the LTGC bias and TRSI/MH-mediated TD in the repair of nCas9-induced DSBs. Although DNA nicks can encounter either the leading strand or the lagging strand of DNA replication, being converted into one-ended DSBs, only the collision with the lagging strand promotes preferential accumulation of nCas9-induced GFP^+^RFP^+^ cells (i.e., non-allelic SCR/LTGC) or GFP^−^RFP^+^ cells (i.e., TRSI/MH-mediated TD) in *BRCA1*-deficient cells. As mutational signatures in human cancer genomes recurrently exhibit a bias towards replication strand^55–57^, our data indicate a replication strand asymmetry in inducing SV mutational signatures in the human cancer genome. Thus, it is of value to determine the contribution of DNA nicks on either replication strand to the mutational processes leading to these asymmetric mutational signatures.

It is unclear how this replication strand bias for LTGC is regulated in HR repair of one-ended DSBs by *BRCA1*, but this regulation may involve distinct *BRCA1* functions in end resection, strand invasion and strand annealing with second ends^8,17,29,58–60^. The end resection function of *BRCA1* generates an extended 3’ ssDNA tail from a blunt DNA end or a DNA end with a short overhang. This DNA end resection involves several cofactors including the Mre11/Rad50/Nbs1 (MRN) complex and CtIP, but is antagonized by 53BP1^14,17^. After recruiting BRCA2 via the interaction with PALB2, BRCA1/PALB2/BRCA2 displace the ssDNA-binding protein RPA from the 3’-ssDNA tail and load RAD51 to form a RAD51 nucleoprotein filament for strand invasion and the ensuing DNA synthesis. However, while HR could be severely disrupted by defects in the end resection of the invading end or the RAD51 loading, it is thought that LTGC bias in *BRCA1*-deficient cells is not promoted by these defects, but by failure to engage the second end for termination of STGC^18,23,24^. This failure can be contributed either to delayed or no availability of the second end at one-ended DSBs or to limited resection of the second end^17^. In the lead collapse, the second end generated by the converging fork may contain a long 3’ ssDNA tail due to discontinuous DNA synthesis on the lagging strand and require no further resection for strand annealing with the displaced nascent strand of DNA synthesis to terminate STGC (Supplementary Fig. 12). In contrast, the second end in the lag collapse is likely a blunt end and requires end resection for strand annealing. Thus, the end section function of *BRCA1* at the second ends could induce a strand asymmetry favoring the lag collapse in suppression of LTGC bias.

Upon encountering difficulties in strand invasion in cells deficient for *BRCA1*, the invading end could engage the second end provided in a later time for NHEJ or a distal end for translocation. Because HR is the primary pathway in the repair of one-ended DSBs in wild-type cells, a shift to local NHEJ or NHEJ-mediated translocation becomes more apparent in HR-defective cells including cells deficient for *BRCA1* or likely *BRCA2* (Supplementary Fig. 12). Unlike BRCA1, BRCA2 is not involved in end resection or the availability of the second end; the reason why BRCA2 suppresses LTGC bias is not clear. It has been proposed that RAD51-independent BIR-type copying mechanism (or RAD51-independent LTGC) may operate in mammalian cells^17^. Thus, the LTGC bias could be elevated in cells deficient for *BRCA2* or *RAD51* where the overall efficiency of HR is reduced. A RAD51-independent pathway may also determine the involvement of BRCA1, not BRCA2 or RAD51, in MH-mediated TD at replication forks stalled by a Tus-Ter replication fork barrier^24^. While BRCA1 suppresses TRSI- and MH-mediated TD in the repair of one-ended DSBs converted from nCas9-induced nicks by DNA replication, it has yet to be determined whether BRCA2 acts in these two mechanisms. However, the involvement of BRCA2 should not be expected since *BRCA2*-mutant cancers do not show typical ~10-kb MH-mediated TDs^4,6^.

In clinical applications of CRISPR/Cas9 genome editing, Cas9-induced two-ended DSBs are considered as a serious safety issue because two-ended DSBs can cause undesired on-target and off-target rearrangements. Two-ended DSBs also induce p53-mediated DDR that suppresses genome editing^61,62^. Consequently, pre-existing inactivation of this pathway is preferentially selected in CRISPR/Cas9 genome editing, potentially increasing a cancer risk^61,62^. In contrast, nCas9-based applications such as base editing and prime editing do not generate two-ended DSBs and are thought to avoid DSB-related safety issues^63–65^. However, as shown in this study, nCas9-induced nicks, in particular in the context of persistent nCas9-sgRNA target residency, can be readily converted into one-ended DSBs by DNA replication, thus activating the DDR and causing chromosomal rearrangements, chromosomal aberrations and micronuclei formation in normal cells. These by-products may become even more prevalent in HR-defective cells such as *BRCA1*-deficient cells. Therefore, besides safety concerns related to Cas9-induced two-ended DSBs, cautions should also be taken in nCas9-based applications in cycling cells. In particular, while stronger target binding or longer target residence duration may help improve the efficiency of nCas9-based genome editing^65^, this strategy may increase the replication-driven conversion of nCas9-induced nicks to one-ended DSBs. The conflicting needs should be balanced in order to improve nCas9-based genome modifications.

## Methods

### Plasmids and chemical reagents

Plasmid px330 was originally obtained from Addgene (Cat #42230) and the SpCas9 cassette was cloned into a pcDNA3β-Hyg-based expression vector. Plasmids expressing nCas9 (D10A and H840A) were generated by site-directed mutation from pcDNA3β-Cas9 using KOD-Plus-Neo Kit (TOYOBO). Plasmids of sgRNAs were constructed from the U6-sgRNA vector as described previously^46^. The sgRNA target sequences are listed in Supplementary Table 1. The newly constructed plasmids were confirmed by Sanger sequencing. Chemical treatments were performed with Aphidicolin (CAS 38966-21-1, Sigma) at 5 μg/mL, L-Mimosine (S7446, Selleck) and Hydroxyurea (S1896, Selleck) at 2 mM, Bleomycin (S1214, Selleck) at 20 μg/mL, Olaparib (S1060, Selleck) at 2 μM and Camptothecin (S1288, Selleck) at 1 μM.

### Cell lines and cell culture

mESC were grown in the DMEM medium supplied with 20% fetal bovine serum (Gibco), 1% penicillin-streptomycin (Gibco), 2 mM L-glutamine (Gibco), 0.1 mM β-mercaptoethanol (Sigma), 0.1 mM non-essential amino acid (Gibco), 1 mM sodium pyruvate (Gibco) and 1000 U/mL leukemia inhibitory factor (Millipore) on either MEF feeders or gelatinized plates. Mouse NIH3T3 cells and human U2OS cells were cultured in high glucose DMEM containing 10% fetal bovine serum, 1% penicillin-streptomycin and 2 mM L-glutamine. *Brca1*-deficient cell lines were generated by deletion of Brca1 BRCT domain using paired Cas9-sgRNAs^34^. Briefly, 2 × 10^5^ mESC were transfected with the expression plasmids for paired sgRNAs and Cas9 in a 24-well plate, and were seeded onto MEF feeder cells at a 10 cm plate for single clones without any antibiotic selection. Single clones were picked, expanded and verified by PCR along with Sanger sequencing and Western blot for *Brca1*-deficient clones. PCR primers were listed in Supplementary Table 2.

### Transfection and reporter assays

Transfection of mESC was performed with Lipofectamine 2000 (Invitrogen) in a 24-well plate as previously described^66,67^. Total 2 × 10^5^ mESC harboring the HR/NHEJ reporter were transfected with 0.5 μg total DNA. For U2OS and NIH3T3 cells, 1.0 × 10^5^ cells were seeded on a 24-well plate and total 0.8 μg DNA were transfected by Lipofectamine 2000. To initiate DNA replication in SCR-RFP reporter U2OS cells, 0.16 μg of the SV40 LT expression plasmid in 0.8 μg of total DNA was co-transfected with expression plasmids for I-SceI or CRISPR nucleases. For the siRNA assay, U2OS cells were transfected with 20 pmol siRNA together with 0.8 μg DNA such as the Cas9/nCas9-sgRNA expression plasmids. For chemical treatment, small molecule inhibitors were added at 6 h post-transfection, and replaced with fresh ones the next day for a continued treatment for the rest of the experiment. Transfected or treated cells were analyzed for *GFP*^*+*^*RFP*^*−*^, *GFP*^*+*^*RFP*^*+*^ and *GFP*^*−*^*RFP*^*+*^ frequencies using the Beckman Coulter CytoFLEX flow cytometer at least 3 days post-transfection. Fluorescence-activated cell sorting (FACS) data were analyzed using the CytExpert 2.0 software. The HR/NHEJ frequencies were calculated after being corrected with background readings and normalized with transfection efficiencies as described before^47,66^.

### Generation of EMX1-SCR reporter mESC

A 138-nt ssODN repair template (Supplementary Table 2) was designed to contain a short sequence of human *EMX1* as additional cleavage sites for Cas9 flanked by homologous sequences from the SCR-RFP reporter and synthesized by TsingKe Biological Technology. The ssODNs along with the Cas9-sgRNA expression plasmids were transfected into mESC and Cas9-induced DSB at specific site in the SCR-RFP reporter allowed site-specific knock-in of short *EMX1* sequence. After 72 h post-transfection, cells were seeded on MEF feeder cells for the formation of single colonies without selection. gDNA was extracted with a purification kit (Axygen), and site-specific knock-in of the short *EMX1* sequence verified by PCR and Sanger sequencing to confirm the establishment of the EMX1-SCR reporter in mESC. *Brca1*-deficient cell lines carrying the EMX1-SCR reporter were generated using the paired Cas9-sgRNA approach as described above.

### Analysis of metaphase spread and micronucleus formation

24 h post-transfection, cells were treated with colchicine (NSC 757, Selleck) at 0.2 μg/mL for 2 h to arrest in metaphase. The cells in metaphase were harvested and washed with cold PBS twice, and then incubated in 0.075 M KCl at 37 °C for 20 min. Hypotonic cells were then centrifuged for 10 min at 1000 rpm and fixed in Cornoy’s fixative (methanol:acetic acid = 3:1) for 20 min at room temperature twice. Cells were resuspended with 0.5 mL Cornoy’s fixative and the suspension was dropped on a lean slide to release chromosomes. These slides were stained with 1% Giemsa solution for 5 min and mounted with neutral balsam (Sangon Biotech). All metaphase spread plates were imaged at a magnification of 1000x using a fluorescence microscope (Leica DM4000). Different types of chromosomal aberrations such as chromatid breaks and radial chromosomes were scored and calculated as percentage of chromosomal aberrations.

For micronucleus preparation, cells were grown on glass coverslips in a 6-well plate and washed gently with cold PBS twice after 24 h post-transfection. Cells were fixed with 4% paraformaldehyde and stained with DAPI. All plates were imaged at a magnification of 1000x using a fluorescence microscope (Leica DM4000). Micronuclei were defined as discrete DNA aggregates juxtaposed to primary nuclei in cells and the frequency of micronucleated cells was calculated.

### Western blot and Immunofluorescence

For western blot, cells were harvested after 24 h post-transfection or 2 h post treatment with chemicals and lysed with RIPA buffer for 30 min. Cell extractions were separated by SDS-PAGE electrophoresis and analyzed by western blot with corresponding antibodies. The following primary antibodies used in this study were anti-Gapdh (EM1101, 1:1000) and anti-RPA32 (ET7109-41, 1:1000) from HuaBio; anti-γH2AX (#05-636, 1:1000) and anti-H2AX (#07-627, 1:1000) from Millipore; anti-p-Chk1 (#2348, 1:1000), anti-p-Chk2 (#2661, 1:1000), anti-p-p53 (#9284, 1:1000), anti-Chk2 (#2662, 1:1000) and anti-p53 (#2524, 1:1000) from Cell Signaling Technology; anti-Chk1 (sc-8408, 1:1000) from Santa Cruz; anti-p-RPA32 (ab87277, 1:1000) and anti-53BP1 (ab21083, 1:500) from Abcam; anti-Brca1 antibody was a gift from the L.Y. Lu Lab^68^.

For immunofluorescence staining, cells were grown on glass coverslips in a 24-well plate and fixed with 4% paraformaldehyde after culturing for 24 h with or without chemical treatment. Cells were permeabilized with 0.1% Triton X-100, followed by blocking in 5% bovine serum albumin in PBS. Cells were probed with primary antibodies and Alexa Fluor 488-conjugated secondary antibodies (#115-585-003, 1:1000) or Alexa Fluor 594-conjugated secondary antibodies (#111-545-003, 1:1000) from Jackson ImmunoResearch, stained with DAPI, and imaged by a fluorescence microscope (Leica DM4000).

### RNA interference and quantitative reverse transcription PCR

Small interference RNAs (siRNAs) targeting human *BRCA1* and a ‘Scramble’ siRNA as control were purchased from RiboBio Co. At 3 days after transfection of 1.0 × 10^5^ U2OS cells with 20 pmol siRNA together with the Cas9/nCas9-sgRNA expression plasmids, RNAs were isolated and reverse-transcribed to complementary DNA using the HiScript II Q RT SuperMix for qPCR (Vazyme). Quantitative reverse transcriptase PCR (qRT-PCR) was performed for siRNA-mediated *BRCA1* depletion on qPCR CFX 96 Thermocycler (Bio-Rad) using gene-specific primers (Supplementary Table 2).

### Cell proliferation assays

Cells were transfected and seeded into a 96-well plate. Cell proliferation activity was measured at indicated days according to the manufacturer’s instructions (CellTiter-AQueous MTS assay, Promega). Briefly, 10 μL of MTS reagents were added directly to the wells, and cell plates were incubated at 37 °C for 1 h. Absorbance was measured at 490 nm on a SpectraMax M5 reader (Molecular Devices).

### PCR amplification and NHEJ quantification by Illumina deep sequencing

To analyze the indel patterns in NHEJ products, cells were collected after NHEJ induced by Cas9 or nCas9. Briefly, at 72 h after cell transfection with expression plasmids for Cas9- or nCas9-sgRNA, cells were harvested and gDNA was isolated as described above. The targeted regions of less than 300 bp in gDNA were PCR-amplified with primers listed in Supplementary Table 2. Next-generation sequencing was performed at Novogene Co. Ltd (Beijing). PCR products purified with PCR Clean-up kit (Axygen) were end-repaired, adenylated at 3′ ends, ligated with adapters, purified, and amplified by the second round of PCR to incorporate the P7 and P5 Illumina adapters according to the manufacturer’s protocols (Yeasen, Hieff NGS Ultima DNA Library Prep Kit for Illumina). Sequences were analyzed to determine the editing efficiency and identify the indel pattern at repair junctions using DBS-Aligner as described previously^46^.

### Detection of translocation events

To detect the intrachromosomal and interchromosomal translocation events, cells were transfected with expression plasmids for Cas9- or nCas9-sgRNA and transfection with pcDNA3β-GFP was used as transfection efficiencies. After 72 h, cells were harvested and gDNA was isolated for PCR. Translocation events with junctions, together with the *Gapdh* region as internal PCR control, were amplified from each sample and mixed in the 10:1 ratio in volume for next-generation sequencing. The relative frequency of each translocation event was calculated as the ratio of translocation reads to *Gapdh* reads and normalized by transfection efficiency. Translocation breakpoints were determined by DBS-Aligner^46^.

### Analysis of TD breakpoints in *RFP*^*+*^ cells

Cells transfected were sorted for spontaneous *RFP*^*+*^ cells and induced *RFP*^*+*^ cells after 72 h transfection by FACS using Beckman Moflo Astrios EQ. Sorted cells were seeded on a gelatinized 6-well plate for expansion and also on MEF feeder cells for single clone isolation. gDNA from each *RFP*^*+*^ single clone was isolated. Breakpoint junctions at the invasion site and the termination site were PCR-amplified with primers listed in Supplementary Table 2 and determined by Sanger sequencing. Breakpoint sequences from individual clones were analyzed and aligned to determine particular TD types.

### Reporting summary

Further information on research design is available in the Nature Research Reporting Summary linked to this article.

## Supplementary information

Supplementary Information

Description of Additional Supplementary Data

Supplementary Data 1

Reporting Summary

## Data Availability

Microscope datasets are available at Zenodo [10.5281/zenodo.6796523]. The raw sequencing datasets that support the findings of this study are available from the NCBI Sequence Read Archive (SRA) with the accession number code PRJNA796450. The annotated nucleotide sequences of the SCR-RFP reporter and the NHEJ reporter were deposited in NCBI Genbank with the accession code ON934620 and ON934621. Source data are provided with this paper.

## Source data

Source Data

## References

[1] Davies H (2017). HRDetect is a predictor of BRCA1 and BRCA2 deficiency based on mutational signatures. Nat. Med..

[2] Lal A (2019). Comprehensive genomic characterization of breast tumors with BRCA1 and BRCA2 mutations. BMC Med. Genomics.

[3] Li Y (2020). Patterns of somatic structural variation in human cancer genomes. Nature.

[4] Menghi F (2018). The Tandem duplicator phenotype is a prevalent genome-wide cancer configuration driven by distinct gene mutations. Cancer Cell.

[5] Nik-Zainal S (2012). Mutational processes molding the genomes of 21 breast cancers. Cell.

[6] Nik-Zainal S (2016). Landscape of somatic mutations in 560 breast cancer whole-genome sequences. Nature.

[7] Zámborszky J (2017). Loss of BRCA1 or BRCA2 markedly increases the rate of base substitution mutagenesis and has distinct effects on genomic deletions. Oncogene.

[8] Chen C-C, Feng W, Lim PX, Kass EM, Jasin M (2018). Homology-directed repair and the role of BRCA1, BRCA2, and related proteins in genome integrity and cancer. Annu. Rev. Cancer Biol..

[9] Stok C, Kok YP, van den Tempel N, van Vugt MATM (2021). Shaping the BRCAness mutational landscape by alternative double-strand break repair, replication stress and mitotic aberrancies. Nucleic Acids Res..

[10] Carvajal-Garcia J (2020). Mechanistic basis for microhomology identification and genome scarring by polymerase theta. Proc. Natl Acad. Sci. USA.

[11] Ceccaldi R (2015). Homologous-recombination-deficient tumours are dependent on Polθ-mediated repair. Nature.

[12] Kamp JA, van Schendel R, Dilweg IW, Tijsterman M (2020). BRCA1-associated structural variations are a consequence of polymerase theta-mediated end-joining. Nat. Commun..

[13] Setton J, Reis-Filho JS, Powell SN (2021). Homologous recombination deficiency: how genomic signatures are generated. Curr. Opin. Genet. Dev..

[14] Mirman Z, de Lange T (2020). 53BP1: a DSB escort. Genes Dev..

[15] Sy SMH, Huen MSY, Chen J (2009). PALB2 is an integral component of the BRCA complex required for homologous recombination repair. Proc. Natl Acad. Sci. USA.

[16] Zhang F (2009). PALB2 links BRCA1 and BRCA2 in the DNA-damage response. Curr. Biol..

[17] Scully R, Panday A, Elango R, Willis NA (2019). DNA double-strand break repair-pathway choice in somatic mammalian cells. Nat. Rev. Mol. Cell Biol..

[18] Chandramouly G (2013). BRCA1 and CtIP suppress long-tract gene conversion between sister chromatids. Nat. Commun..

[19] Carvalho CMB, Lupski JR (2016). Mechanisms underlying structural variant formation in genomic disorders. Nat. Rev. Genet..

[20] Epum EA, Haber JE (2022). DNA replication: the recombination connection. Trends Cell Biol..

[21] Costantino L (2014). Break-induced replication repair of damaged forks induces genomic duplications in human cells. Science.

[22] Wu X, Malkova A (2021). Break-induced replication mechanisms in yeast and mammals. Curr. Opin. Genet. Dev..

[23] Willis NA (2014). BRCA1 controls homologous recombination at Tus/Ter-stalled mammalian replication forks. Nature.

[24] Willis NA (2017). Mechanism of tandem duplication formation in BRCA1-mutant cells. Nature.

[25] Caldecott KW (2008). Single-strand break repair and genetic disease. Nat. Rev. Genet..

[26] Li S (2021). PIF1 helicase promotes break-induced replication in mammalian cells. EMBO J..

[27] Nielsen I (2009). A Flp-nick system to study repair of a single protein-bound nick in vivo. Nat. Methods.

[28] Strumberg D (2000). Conversion of topoisomerase I cleavage complexes on the leading strand of ribosomal DNA into 5’-phosphorylated DNA double-strand breaks by replication runoff. Mol. Cell Biol..

[29] Vrtis KB (2021). Single-strand DNA breaks cause replisome disassembly. Mol. Cell.

[30] Jinek M (2012). A programmable dual-RNA-guided DNA endonuclease in adaptive bacterial immunity. Science.

[31] Cong L (2013). Multiplex genome engineering using CRISPR/Cas systems. Science.

[32] Knight SC (2015). Dynamics of CRISPR-Cas9 genome interrogation in living cells. Science.

[33] Sternberg SH, LaFrance B, Kaplan M, Doudna JA (2015). Conformational control of DNA target cleavage by CRISPR-Cas9. Nature.

[34] Guo T (2018). Harnessing accurate non-homologous end joining for efficient precise deletion in CRISPR/Cas9-mediated genome editing. Genome Biol..

[35] Williams RS (2003). Detection of protein folding defects caused by BRCA1-BRCT truncation and missense mutations. J. Biol. Chem..

[36] Liu L (2021). Tracking break-induced replication shows that it stalls at roadblocks. Nature.

[37] Clarke R (2018). Enhanced Bacterial immunity and mammalian genome editing via RNA-polymerase-mediated dislodging of Cas9 from double-strand DNA breaks. Mol. Cell.

[38] Doi G (2021). Catalytically inactive Cas9 impairs DNA replication fork progression to induce focal genomic instability. Nucleic Acids Res.

[39] Laughery MF, Mayes HC, Pedroza IK, Wyrick JJ (2019). R-loop formation by dCas9 is mutagenic in Saccharomyces cerevisiae. Nucleic Acids Res.

[40] Xu X (1999). Centrosome amplification and a defective G2-M cell cycle checkpoint induce genetic instability in BRCA1 exon 11 isoform-deficient cells. Mol. Cell.

[41] Kadyk LC, Hartwell LH (1992). Sister chromatids are preferred over homologs as substrates for recombinational repair in Saccharomyces cerevisiae. Genetics.

[42] Johnson RD, Jasin M (2000). Sister chromatid gene conversion is a prominent double-strand break repair pathway in mammalian cells. EMBO J..

[43] Rass E, Chandramouly G, Zha S, Alt FW, Xie A (2013). Ataxia telangiectasia mutated (ATM) is dispensable for endonuclease I-SceI-induced homologous recombination in mouse embryonic stem cells. J. Biol. Chem..

[44] Truong LN (2014). Homologous recombination is a primary pathway to repair DNA double-strand breaks generated during DNA rereplication. J. Biol. Chem..

[45] Richardson CD, Ray GJ, DeWitt MA, Curie GL, Corn JE (2016). Enhancing homology-directed genome editing by catalytically active and inactive CRISPR-Cas9 using asymmetric donor DNA. Nat. Biotechnol..

[46] Feng Y-L (2017). H2AX facilitates classical non-homologous end joining at the expense of limited nucleotide loss at repair junctions. Nucleic Acids Res..

[47] Xie A, Kwok A, Scully R (2009). Role of mammalian Mre11 in classical and alternative nonhomologous end joining. Nat. Struct. Mol. Biol..

[48] Panday A (2021). FANCM regulates repair pathway choice at stalled replication forks. Mol. Cell.

[49] Puget N, Knowlton M, Scully R (2005). Molecular analysis of sister chromatid recombination in mammalian cells. DNA Repair (Amst.).

[50] Feng Y, Liu S, Chen R, Xie A (2021). Target binding and residence: a new determinant of DNA double-strand break repair pathway choice in CRISPR/Cas9 genome editing. J. Zhejiang Univ. Sci. B.

[51] Sternberg SH, Redding S, Jinek M, Greene EC, Doudna JA (2014). DNA interrogation by the CRISPR RNA-guided endonuclease Cas9. Nature.

[52] Qi LS (2013). Repurposing CRISPR as an RNA-guided platform for sequence-specific control of gene expression. Cell.

[53] Malkova A, Haber JE (2012). Mutations arising during repair of chromosome breaks. Annu. Rev. Genet..

[54] Smith CE, Llorente B, Symington LS (2007). Template switching during break-induced replication. Nature.

[55] Haradhvala NJ (2016). Mutational strand asymmetries in cancer genomes reveal mechanisms of DNA damage and repair. Cell.

[56] Morganella S (2016). The topography of mutational processes in breast cancer genomes. Nat. Commun..

[57] Aitken SJ (2020). Pervasive lesion segregation shapes cancer genome evolution. Nature.

[58] Ira G, Haber JE (2002). Characterization of RAD51-independent break-induced replication that acts preferentially with short homologous sequences. Mol. Cell Biol..

[59] Jakobsen KP (2019). Minimal resection takes place during break-induced replication repair of collapsed replication forks and is controlled by strand invasion. Cell Rep..

[60] Nacson J (2020). BRCA1 mutational complementation induces synthetic viability. Mol. Cell.

[61] Haapaniemi E, Botla S, Persson J, Schmierer B, Taipale J (2018). CRISPR-Cas9 genome editing induces a p53-mediated DNA damage response. Nat. Med..

[62] Ihry RJ (2018). p53 inhibits CRISPR-Cas9 engineering in human pluripotent stem cells. Nat. Med..

[63] Anzalone AV (2019). Search-and-replace genome editing without double-strand breaks or donor DNA. Nature.

[64] Komor AC, Kim YB, Packer MS, Zuris JA, Liu DR (2016). Programmable editing of a target base in genomic DNA without double-stranded DNA cleavage. Nature.

[65] Anzalone AV, Koblan LW, Liu DR (2020). Genome editing with CRISPR-Cas nucleases, base editors, transposases and prime editors. Nat. Biotechnol..

[66] Xie A (2004). Control of sister chromatid recombination by histone H2AX. Mol. Cell.

[67] Willis NA, Scully R (2021). Measurement of homologous recombination at stalled mammalian replication forks. Methods Mol. Biol..

[68] Wu J (2009). Histone ubiquitination associates with BRCA1-dependent DNA damage response. Mol. Cell Biol..

