# Supplementary material for: DNA nicks induce mutational signatures associated with BRCA1 deficiency
Source: Nat Commun. 2022 Jul 25;13:4285. doi: 10.1038/s41467-022-32011-x (PMC9314409; doi:10.1038/s41467-022-32011-x)
Supplement: Supplementary file 1 — Supplementary Information [file 41467_2022_32011_MOESM1_ESM.pdf]

DNA nicks induce mutational signatures associated with *BRCA1* deficiency

Yi-Li Feng[1,2,†,*], Qian Liu[1,2,†], Ruo-Dan Chen[1,2], Si-Cheng Liu[1,2], Zhi-Cheng Huang[1,2], Kun-Ming Liu[1,2], Xiao-Ying Yang[1,2], An-Yong Xie[1,2,*]

[1] Innovation Center for Minimally Invasive Technique and Device, Department of General Surgery, Sir Run Run Shaw Hospital, Zhejiang University School of Medicine, Hangzhou, Zhejiang 310019, P. R. China
[2] Institute of Translational Medicine, Zhejiang University School of Medicine and Zhejiang University Cancer Center, Hangzhou, Zhejiang 310029, P. R. China

[*]Corresponding author (lead contact): Tel: +86 0571 86971680; Fax: +86 571 88981576; Email: anyongxie@zju.edu.cn (An-Yong Xie); eric_feng@zju.edu.cn (Yi-Li Feng)
[†]These authors contributed equally to this work

SUPPLEMENTARY INFORMATION

Supplementary Figures 1-12

Supplementary Tables 1-2

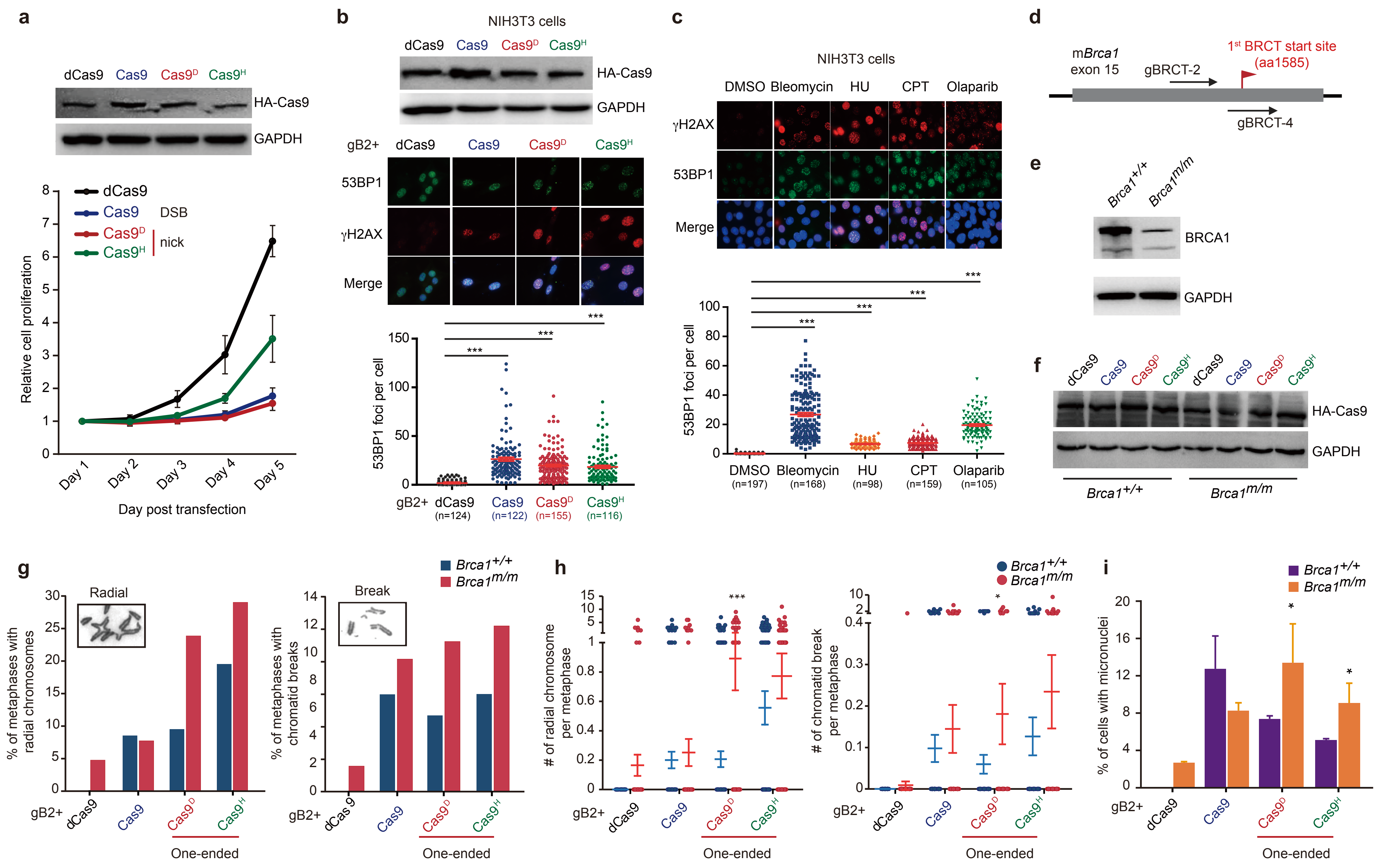

a
dCas9 Cas9 Cas9D Cas9H
HA-Cas9
GAPDH
dCas9
Cas9 DSB
Cas9D nick
Cas9H
Relative cell proliferation
Day 1 Day 2 Day 3 Day 4 Day 5
Day post transfection
b
NIH3T3 cells
dCas9 Cas9 Cas9D Cas9H
HA-Cas9
GAPDH
gB2+ dCas9 Cas9 Cas9D Cas9H
53BP1
γH2AX
Merge
53BP1 foci per cell
gB2+ dCas9 (n=124) Cas9 (n=122) Cas9D (n=155) Cas9H (n=116)
c
NIH3T3 cells
DMSO Bleomycin HU CPT Olaparib
γH2AX
53BP1
Merge
53BP1 foci per cell
DMSO (n=197) Bleomycin (n=168) HU (n=98) CPT (n=159) Olaparib (n=105)
d
mBrca1 exon 15
gBRCT-2
1st BRCT start site (aa1585)
gBRCT-4
e
Brca1+/+ Brca1m/m
BRCA1
GAPDH
f
dCas9 Cas9 Cas9D Cas9H dCas9 Cas9 Cas9D Cas9H
HA-Cas9
GAPDH
Brca1+/+ Brca1m/m
g
Radial
% of metaphases with radial chromosomes
Break
% of metaphases with chromatid breaks
Brca1+/+
Brca1m/m
gB2+ dCas9 Cas9 Cas9D Cas9H
One-ended
h
# of radial chromosome per metaphase
# of chromatid break per metaphase
Brca1+/+
Brca1m/m
gB2+ dCas9 Cas9 Cas9D Cas9H
One-ended
i
% of cells with micronuclei
Brca1+/+
Brca1m/m
gB2+ dCas9 Cas9 Cas9D Cas9H
One-ended

Supplementary Figure 1. nCas9-induced nicks are converted to DSBs and stimulate chromosomal aberrations more in *Brca1*$^{m/m}$ cells than *Brca1*$^{+/+}$ cells. a Effect of Cas9- and nCas9-induced DNA breaks on cell proliferation. mESC were transfected with expression plasmids for Cas9-gB2 and nCas9-gB2 as well as dCas9-gB2 as control and seeded into a 96-well plate. Cell proliferation was analyzed by MTS assay and proliferation relative to Day 1 calculated. The efficiencies of transfection with dCas9, Cas9, Cas9^{D} and Cas9^{H} expression plasmids were similar in each independent set of experiments around 70% and the steady-state levels of these proteins synthesized were shown on top. **b,c** Representative images of 53BP1 and γH2AX focus formation in mouse embryonic fibroblast (MEF) NIH3T3 cells transfected with expression plasmids for dCas9-gB2, Cas9-gB2, Cas9^{D}-gB2 and Cas9^{H}-gB2 (**b**) or treated with DNA damage-inducing agents (**c**) as indicated. Quantification of 53BP1 foci is shown under micrograph. Each dot represents the number of 53BP1 foci in each cell. The efficiencies of transfection with dCas9, Cas9, Cas9^{D} and Cas9^{H} expression plasmids were similar in each independent set of experiments around 70% and the steady-state level of Cas9 and its variants is shown on top. **d** Schematic of precise 49-bp deletion within *Brca1* exon 15 by paired Cas9-sgRNAs in mESC in both alleles, generating BRCA1 lacking C-terminal BRCT domain. **e** Western blot of BRCA1 confirming generation *Brca1*$^{m/m}$ mESC as well as isogenic *Brca1*$^{+/+}$ mESC with Gapdh as loading control. **f** Western blot of HA-tagged Cas9 and its variants expressed in *Brca1*$^{+/+}$ and *Brca1*$^{m/m}$ cells. The efficiencies of transfection with dCas9, Cas9, Cas9^{D} and Cas9^{H} expression plasmids were similar around 70%. **g** Frequencies of metaphases with radial chromosomes (left) and chromatid breaks (right) in *Brca1*$^{+/+}$ and *Brca1*$^{m/m}$ cells. **h** Number of radial chromosomes (left) and chromatid breaks (right) in each metaphase in *Brca1*$^{+/+}$ and *Brca1*$^{m/m}$ cells. One dot indicates one metaphase. **i** Percentages of cells containing micronuclei between *Brca1*$^{+/+}$ and *Brca1*$^{m/m}$ cells. Statistics is performed by unpaired t test with Welch's correction in **b**, **c** and **e**, and by two-tailed Student's t-test in **i**. *, $P<0.05$; **, $P<0.01$; ***, $P<0.001$.

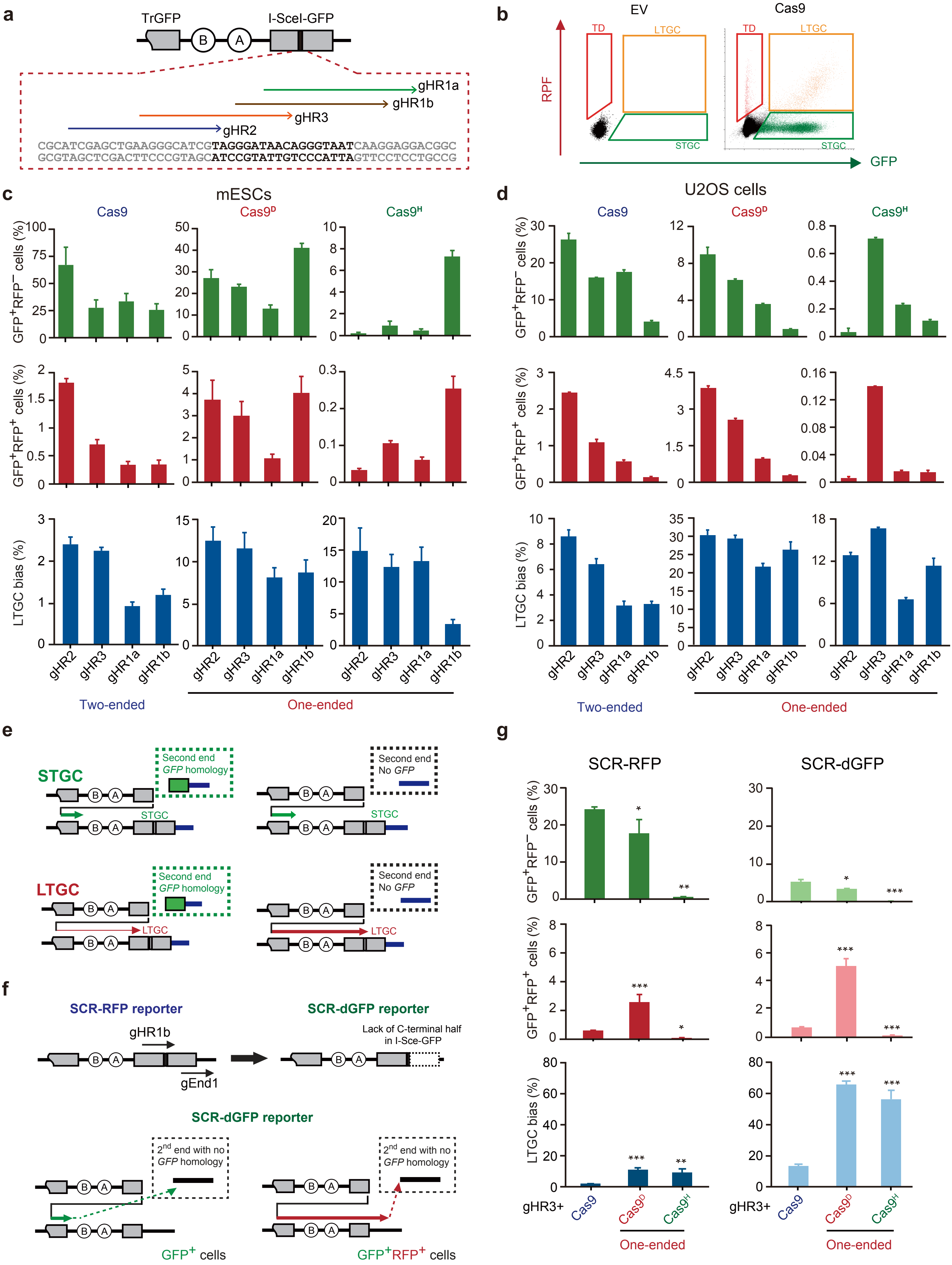

a
TrGFP
B
A
I-SceI-GFP
gHR1a
gHR1b
gHR3
gHR2
CGCATCGAGCTGAAGGGCATCGTAGGGATAACAGGGTAATCAAGGAGGACGGC
GCGTAGCTCGACTTCCCGTAGCATCCCTATTGTCCCATTAGTTCCTCCTGCCG
b
EV
Cas9
TD
LTGC
STGC
RPF
GFP
c
mESCs
Cas9
Cas9D
Cas9H
GFP+RFP− cells (%)
GFP+RFP+ cells (%)
LTGC bias (%)
gHR2
gHR3
gHR1a
gHR1b
Two-ended
One-ended
d
U2OS cells
Cas9
Cas9D
Cas9H
GFP+RFP− cells (%)
GFP+RFP+ cells (%)
LTGC bias (%)
gHR2
gHR3
gHR1a
gHR1b
Two-ended
One-ended
e
STGC
Second end GFP homology
Second end No GFP
STGC
LTGC
Second end GFP homology
Second end No GFP
LTGC
f
SCR-RFP reporter
SCR-dGFP reporter
gHR1b
gEnd1
Lack of C-terminal half in I-Sce-GFP
SCR-dGFP reporter
2nd end with no GFP homology
2nd end with no GFP homology
GFP+ cells
GFP+RFP+ cells
g
SCR-RFP
SCR-dGFP
GFP+RFP− cells (%)
GFP+RFP+ cells (%)
LTGC bias (%)
gHR3+
Cas9
Cas9D
Cas9H
One-ended

Supplementary Figure 2. Lack of GFP homology in the second end promotes LTGC in nCas9-induced HR. a Target sequences for 4 sgRNAs around the I-SceI site of the SCR-RFP reporter. Target sequences are indicated by arrowed lines and the I-SceI site in bold. **b, c** Cas9- or nCas9-induced STGC (top), LTGC (middle) and LTGC bias (bottom) at four independent sites of the SCR-RFP reporter in mESC (**b**) and U2OS cells (**c**). **d** Model for STGC termination by the second ends generated by converging DNA replication forks in repair of one-ended DSBs. In the SCR-RFP reporter, GFP homology in the second end terminates GC in favor of STGC (left). Lack of GFP homology in the second end may extend DNA synthesis in GC until homologous sequence is provided by the second end, thus promoting the LTGC bias (right). **e** Generation of the SCR-dGFP reporter in which the second part of I-Sce-GFP after I-SceI site is deleted by paired Cas9-sgRNAs as indicated in mESC. Due to lack of GFP homology in the second end, LTGC bias is expected to increase. **f** STGC (top), LTGC (middle) and LTGC bias (bottom) induced by Cas9-gHR3 and nCas9-gHR3 in SCR-RFP reporter mESC and SCR-dGFP reporter mESC. Columns indicate the mean ± S.E.M from three independent experiments and statistics is performed by two-tailed Student's t-test in **f**.

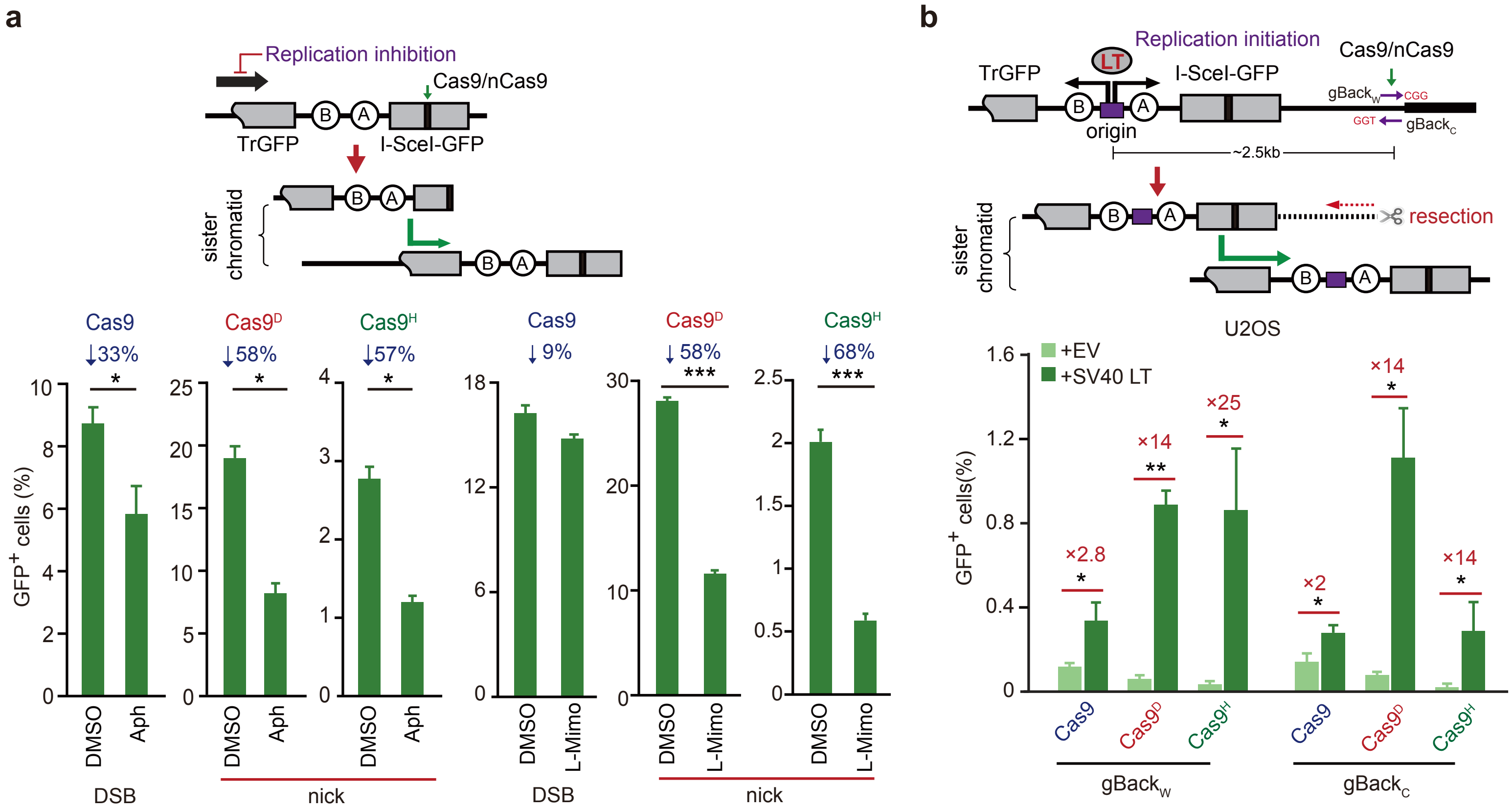

Supplementary Figure 3. nCas9-induced HR is associated with DNA replication. a Effect of Aphidicolin and L-Mimosine on Cas9- or nCas9-induced HR. mESC carrying SCR-RFP reporter (top) were transfected with expression plasmids for Cas9-gHR1b or nCas9-gHR1b and treated with Aphidicolin (0.4 μM) or L-Mimosine (0.5 mM) at 6 h post transfection. Cas9- and nCas9-induced HR was measured by FACS 72 h post transfection. Aphidicolin: Aphi; L-Mimosine, L-Mimo. **b** Impact of local replication on Cas9- or nCas9-induced HR. U2OS cells carrying the SCR-RFP reporter were transfected with expression plasmids for 0.016 μg SV40 LT (1/50 of total plasmid amount transfected) together with Cas9-gBack$_W$, Cas9-gBack$_C$, nCas9-gBack$_W$ or nCas9-gBack$_C$. The gBack$_W$ and gBack$_C$ target sites as indicated are located downstream of *I-Sce-GFP* in the SCR-RFP reporter, about 2.5 kb away from the SV40 origin. SV40 LT binds to the SV40 origin in the SCR-RFP reporter to initiate replication while Cas9 or nCas9 is inducing a break 2.5 kb down-stream of the SV40 origin. Up to 750-nt resection of the end over the I-SceI site allows homologous strand invasion into *TrGFP* of sister chromatid for non-allelic HR as indicated on top diagram. Cas9- and nCas9-induced HR was measured by FACS 72 h post transfection as shown in the bottom panel. Columns indicate the mean ± S.E.M from three independent experiments and statistics is performed by two-tailed Student's t-test. *, P<0.05; **, P<0.01; ***, P<0.001.

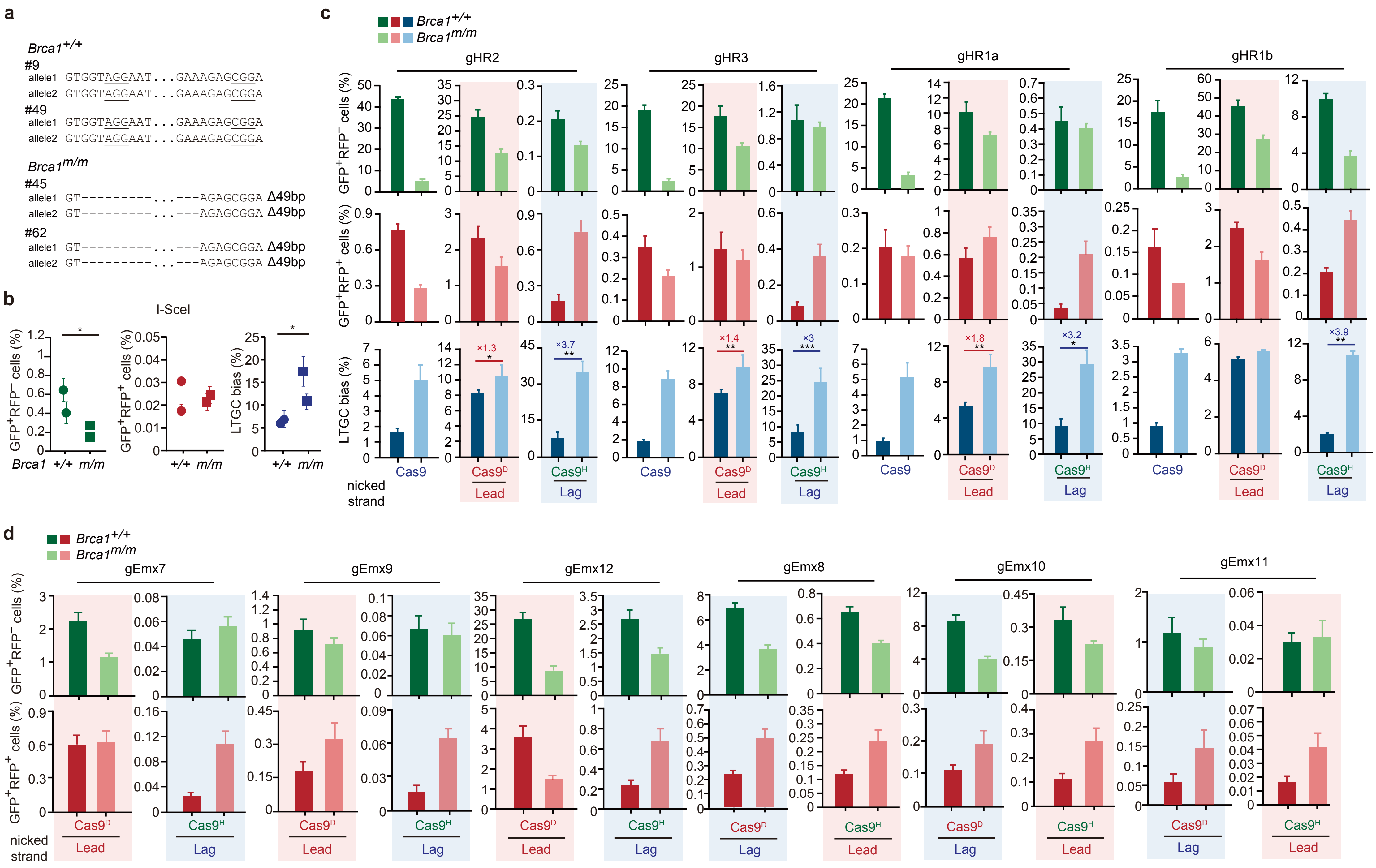
a
Brca1+/+
#9
allele1 GTGGTAGGAAT...GAAAGAGCGGA
allele2 GTGGTAGGAAT...GAAAGAGCGGA
#49
allele1 GTGGTAGGAAT...GAAAGAGCGGA
allele2 GTGGTAGGAAT...GAAAGAGCGGA
Brca1m/m
#45
allele1 GT---------...---AGAGCGGA Δ49bp
allele2 GT---------...---AGAGCGGA Δ49bp
#62
allele1 GT---------...---AGAGCGGA Δ49bp
allele2 GT---------...---AGAGCGGA Δ49bp
b
I-SceI
GFP+RFP− cells (%)
GFP+RFP+ cells (%)
LTGC bias (%)
Brca1 +/+ m/m
c
Brca1+/+
Brca1m/m
gHR2
gHR3
gHR1a
gHR1b
GFP+RFP− cells (%)
GFP+RFP+ cells (%)
LTGC bias (%)
×1.3
×3.7
×1.4
×3
×1.8
×3.2
×3.9
Cas9
Cas9D
Lead
Cas9H
Lag
nicked strand
d
Brca1+/+
Brca1m/m
gEmx7
gEmx9
gEmx12
gEmx8
gEmx10
gEmx11
GFP+RFP− cells (%)
GFP+RFP+ cells (%)
Cas9D
Lead
Cas9H
Lag
nicked strand

Supplementary Figure 4. The effect of *Brca1* deficiency on I-SceI- or Cas9-induced HR. a Generation of SCR-RFP reporter *Brca1*$^{m/m}$ mESC by 49-bp targeted deletion in exon 15 of *Brca1* using paired Cas9-sgRNAs. Isogenic *Brca1*$^{+/+}$ and *Brca1*$^{m/m}$ cells, two clones of each genotype, were confirmed by Sanger sequencing. **b** I-SceI-induced STGC (left), LTGC (middle) and LTGC bias (right) in isogenic *Brca1*$^{+/+}$ and *Brca1*$^{m/m}$ mESC clones, two each genotype. **c,d** Frequencies of STGC (top), LTGC (middle) and LTGC bias (bottom) induced by Cas9 or nCas9 together with each of 4 indicated sgRNAs in the SCR-RFP reporter (**c**) and with each of 6 indicated sgRNAs in the EMX1-SCR reporter (**d**) in isogenic *Brca1*$^{+/+}$ and *Brca1*$^{m/m}$ mESC. The replication strand that encounters nCas9-induced nicks, potentially leading to LTGC products (i.e., GFP^+RFP^+ cells), is indicated. Each symbol represents the mean of at least three independent experiments for single clones and statistics is performed by One-way ANOVA with Tukey's multiple comparison test in **b**. Columns indicate the mean ± S.E.M from three independent experiments and statistics is performed by two-tailed Student's t-test in **c** and **d**. *, P<0.05; **, P<0.01; ***, P<0.001.

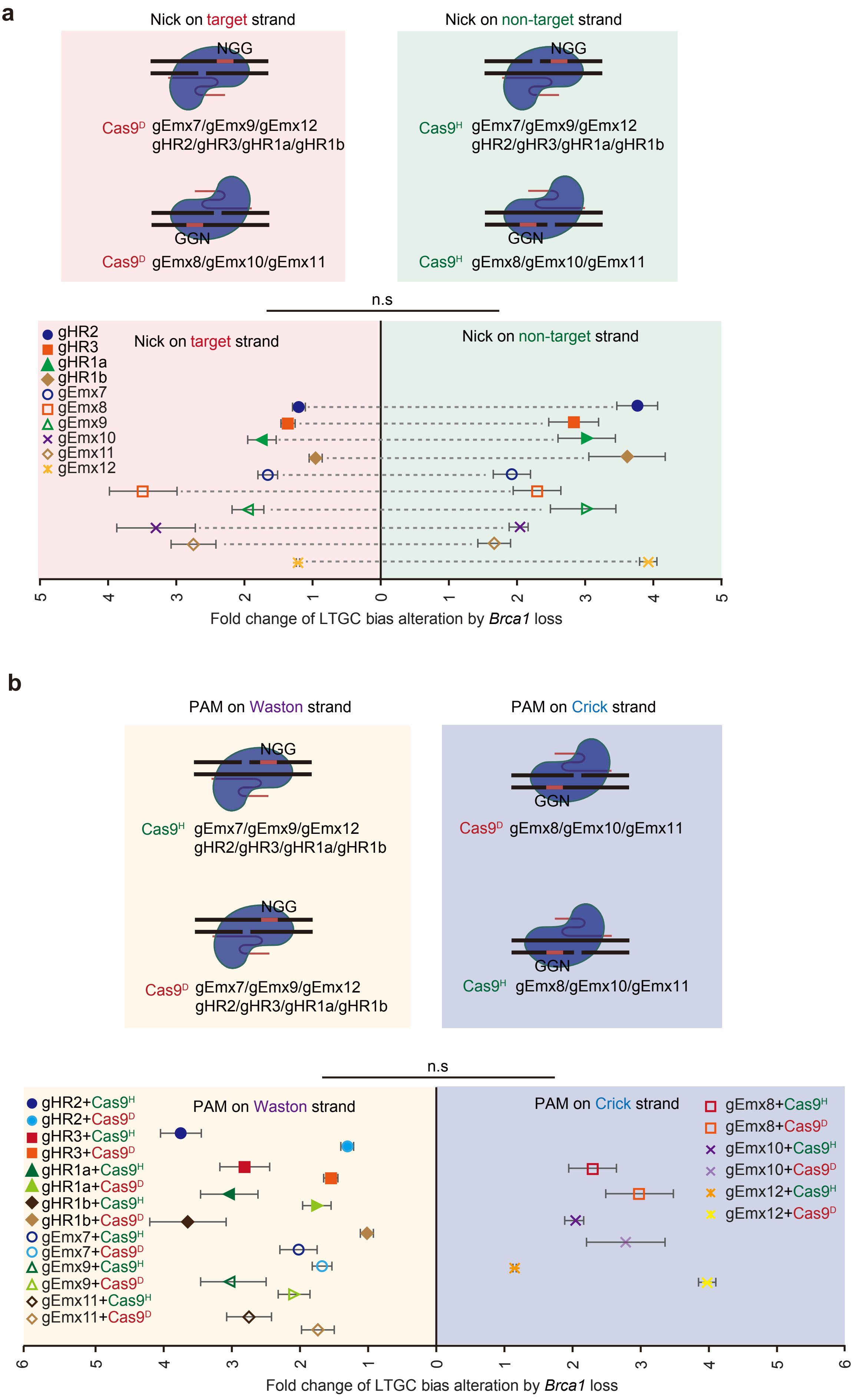
a
Nick on target strand
NGG
Cas9^{D} gEmx7/gEmx9/gEmx12
gHR2/gHR3/gHR1a/gHR1b
GGN
Cas9^{D} gEmx8/gEmx10/gEmx11
Nick on non-target strand
NGG
Cas9^{H} gEmx7/gEmx9/gEmx12
gHR2/gHR3/gHR1a/gHR1b
GGN
Cas9^{H} gEmx8/gEmx10/gEmx11
n.s
gHR2
gHR3
gHR1a
gHR1b
gEmx7
gEmx8
gEmx9
gEmx10
gEmx11
gEmx12
Nick on target strand
Nick on non-target strand
5 4 3 2 1 0 1 2 3 4 5
Fold change of LTGC bias alteration by *Brca1* loss
b
PAM on Waston strand
NGG
Cas9^{H} gEmx7/gEmx9/gEmx12
gHR2/gHR3/gHR1a/gHR1b
NGG
Cas9^{D} gEmx7/gEmx9/gEmx12
gHR2/gHR3/gHR1a/gHR1b
PAM on Crick strand
GGN
Cas9^{D} gEmx8/gEmx10/gEmx11
GGN
Cas9^{H} gEmx8/gEmx10/gEmx11
n.s
gHR2+Cas9^{H}
gHR2+Cas9^{D}
gHR3+Cas9^{H}
gHR3+Cas9^{D}
gHR1a+Cas9^{H}
gHR1a+Cas9^{D}
gHR1b+Cas9^{H}
gHR1b+Cas9^{D}
gEmx7+Cas9^{H}
gEmx7+Cas9^{D}
gEmx9+Cas9^{H}
gEmx9+Cas9^{D}
gEmx11+Cas9^{H}
gEmx11+Cas9^{D}
PAM on Waston strand
PAM on Crick strand
gEmx8+Cas9^{H}
gEmx8+Cas9^{D}
gEmx10+Cas9^{H}
gEmx10+Cas9^{D}
gEmx12+Cas9^{H}
gEmx12+Cas9^{D}
6 5 4 3 2 1 0 1 2 3 4 5 6
Fold change of LTGC bias alteration by *Brca1* loss

Supplementary Figure 5. Conformational context of the Cas9-sgRNA-DNA complex has no effect on BRCA1-mediated suppression of LTGC bias. a *Brca1* deficiency-mediated alteration of LTGC bias for nicks induced on target DNA strand in the DNA-RNA hybrid and on non-target DNA strand. Conformational contexts of nicked strands in the Cas9-sgRNA-DNA complex are shown on top. **b** *Brca1* deficiency-mediated alteration of LTGC bias for nicks induced by Cas9-sgRNA with PAM on the Watson strand and on the Crick strand. The PAM position for the Cas9-sgRNA-DNA complex that induces nicks is shown on top. Each symbol represents the mean of at least three independent experiments for one sgRNA, and two-tailed Student's t-test is performed. ns, P>0.05.

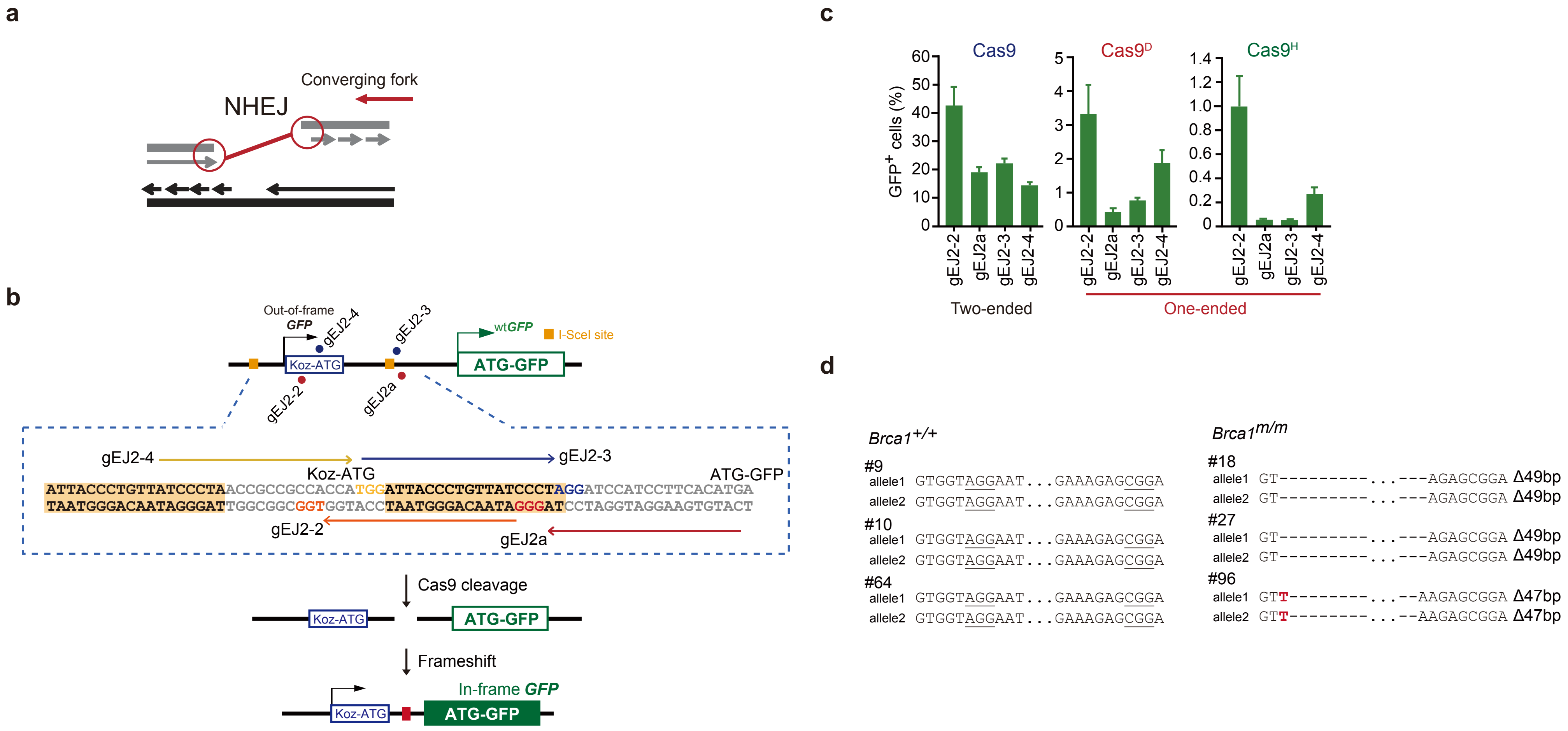

Supplementary Figure 6. Simultaneous presence of the second end is limited for NHEJ of one-ended DSBs. a Local NHEJ of nCas9-induced one-ended DSBs due to generation of the second end from the converging DNA replication fork. **b** Schematic of the NHEJ reporter containing a *GFP* expression cassette driven by the *PGK* promoter. Normally, no in-frame GFP is translated due to an upstream, out-of-frame translation start site Kozak-ATG ('Koz-ATG') flanked by two sequentially positioned I-SceI sites. Upon DNA breakage induced by Cas9 or nCas9 at each of four independent sgRNA targets around the second I-SceI site as indicated, NHEJ repair of two-ended and one-ended DSBs can induce indels between Koz-ATG and ATG-GFP. Some of these indels can lead to in-frame GFP by reframing in a certain probability. Thus, the frequency of GFP^+ cells generated represents the level of NHEJ in cells. Arrowed lines indicate sgRNA targets with the arrow pointing to the NGG PAM. **c** Comparison between Cas9- and nCas9-induced NHEJ in NHEJ reporter mESC with the four targeting sgRNAs as indicated. **d** Generation of NHEJ reporter *Brca1*$^{m/m}$ mESC by 49-bp targeted deletion in exon 15 of *Brca1* using paired Cas9-sgRNAs. Isogenic *Brca1*$^{+/+}$ and *Brca1*$^{m/m}$ cells, three clones of each genotype, were confirmed by Sanger sequencing.

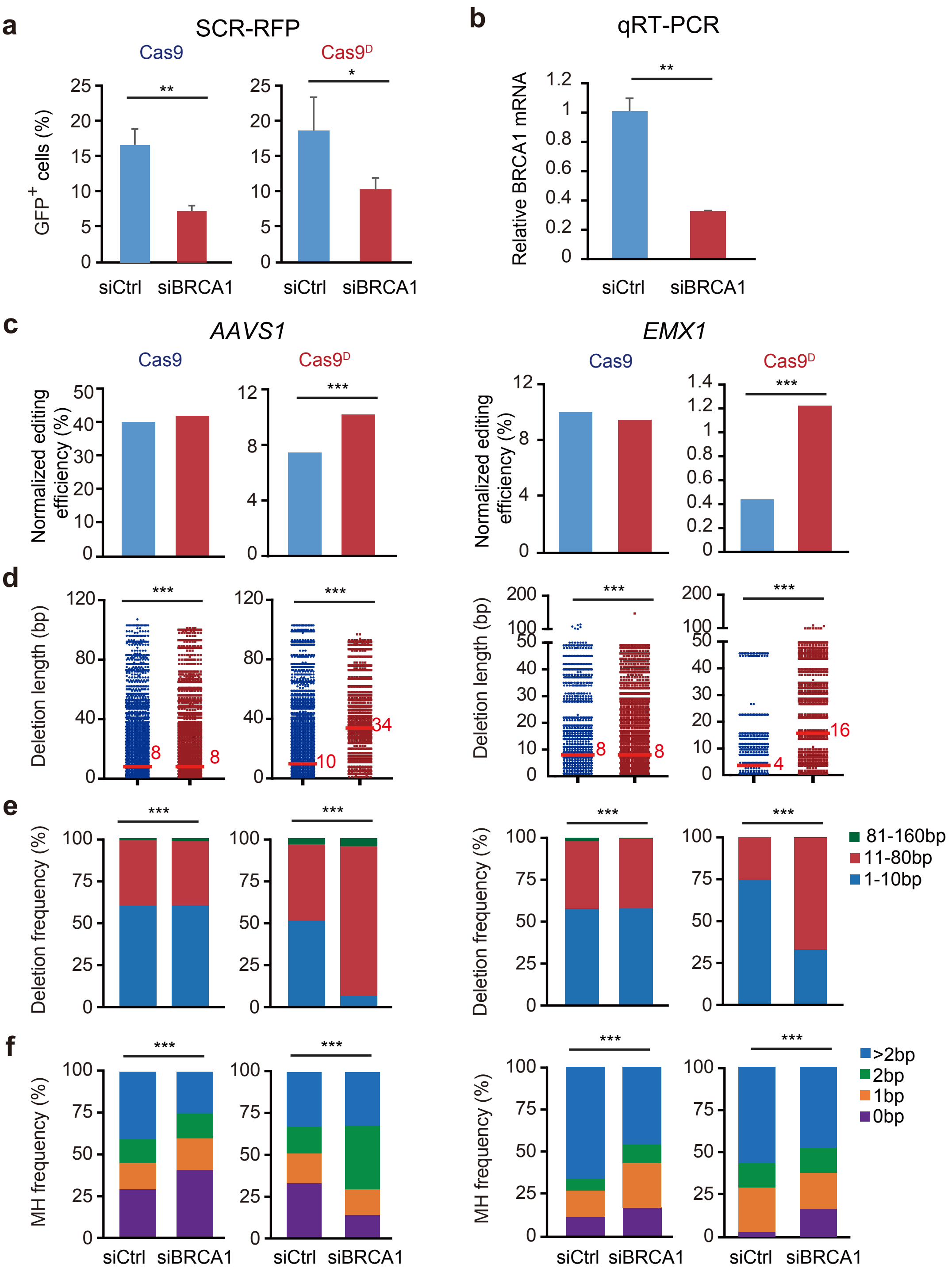

Supplementary Figure 7. *BRCA1* depletion stimulates nick-induced NHEJ with longer deletions and more MH usage at repair junctions in U2OS cells. a Cas9- or nCas9-induced HR in SCR-RFP reporter U2OS cells depleted and not depleted of *BRCA1* (i.e., siBRCA1 vs. siCtrl). **b** siRNA-mediated depletion of *BRCA1* in U2OS cells detected by qRT-PCR at 3 days post transfection with siRNA. **c** Cas9- or nCas9-induced NHEJ at the intron 2 of the *AAVS1* locus and the intron 2 of the *EMX1* locus in U2OS cells depleted and not depleted of *BRCA1*. The NHEJ-mediated editing efficiency is calculated as ratios of edited reads to total reads from targeted Illumina sequencing and normalized by respective transfection efficiency. **d-f**, Analysis of NHEJ junctions for deletion length with the median length indicated (**c**), distribution of deletions with three size ranges (**d**) and the usage of MH (**e**) between U2OS cells depleted and not depleted of *BRCA1*. Columns indicate the mean ± S.E.M from three independent experiments and statistics is performed by two-tailed Student's t-test in **a**. The numbers of edited reads and total reads in **c** include: 64715/363981 for siCtrl and 94939/484514 for siBRCA1 in Cas9-induced NHEJ, and 30694/885856 for siCtrl and 33835/651904 for siBRCA1 in Cas9^D-induced NHEJ at the *AAVS1* site; and 19779/448947 for siCtrl and 36270/851907 for siBRCA1 in Cas9-induced NHEJ, and 1570/952252 for siCtrl and 5956/1094855 for siBRCA1 in Cas9^D-induced NHEJ at the *EMX1* site. Statistical significance is detected by χ2 test in **c**. Statistics is performed on the edited reads by two-tailed Mann–Whitney test in **d-f**. *, P<0.05; **, P<0.01 and ***, P<0.001.

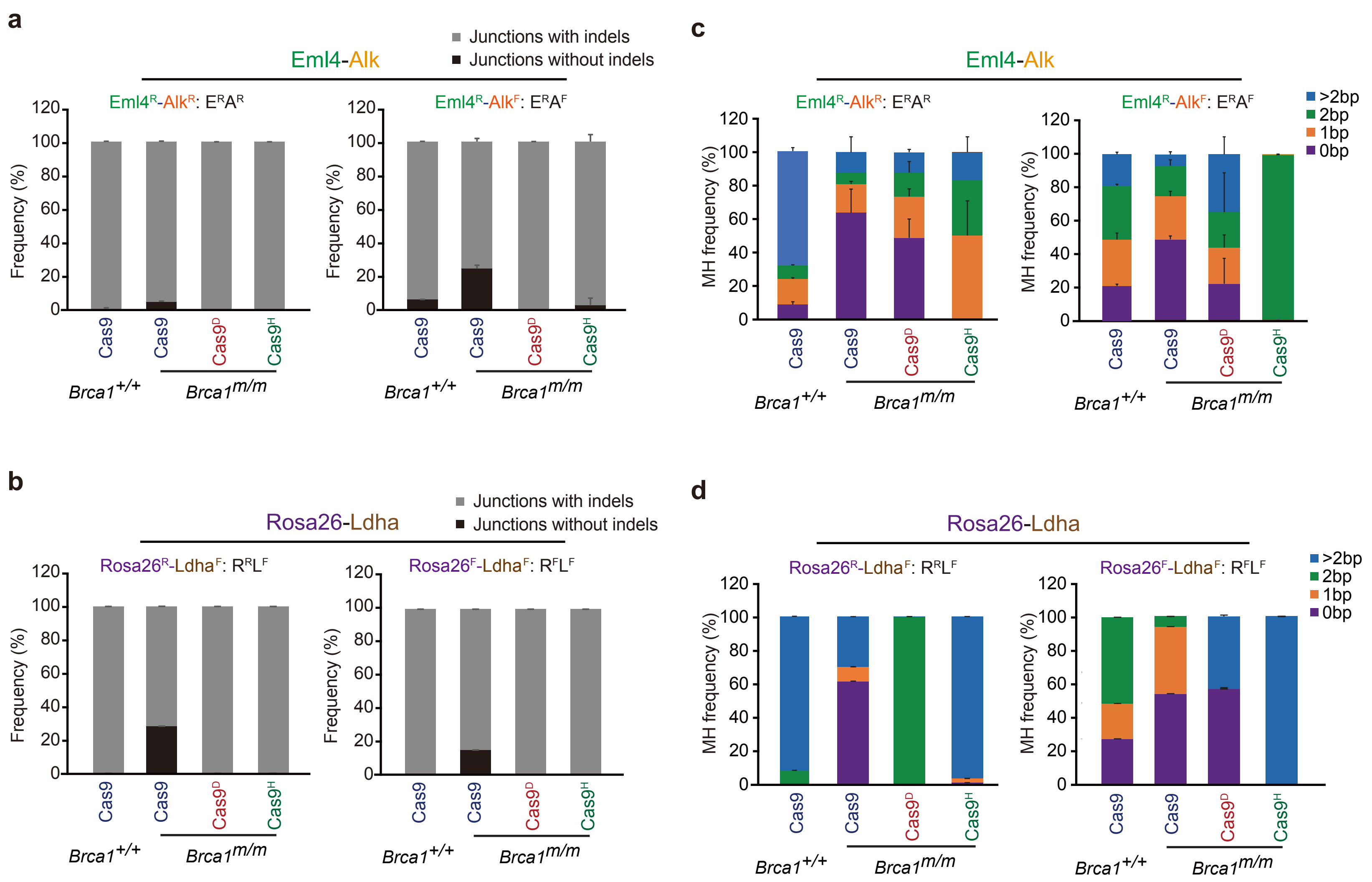

Supplementary Figure 8. Analysis of translocation junctions in *Brca1*$^{+/+}$ and *Brca1*$^{m/m}$ mESC. a, b Frequencies of junctions with or without indel in *Eml4–Alk* translocations E^RA^R and E^RA^F (**a**) and *Rosa26–Ldha* translocations R^RL^F and R^FL^F (**b**) between *Brca1*$^{+/+}$ *and Brca1*$^{m/m}$ mESC. Columns indicate the mean ± S.E.M from three independent experiments. **c, d** Usage of MH at the junctions of *Eml4–Alk* translocations E^RA^R and E^RA^F (**c**) and *Rosa26–Ldha* translocations R^RL^F and R^FL^F (**d**) between *Brca1*$^{+/+}$ *and Brca1*$^{m/m}$ mESC. Only junctions with deletions are included. Columns indicate the mean ± S.E.M from three independent experiments.

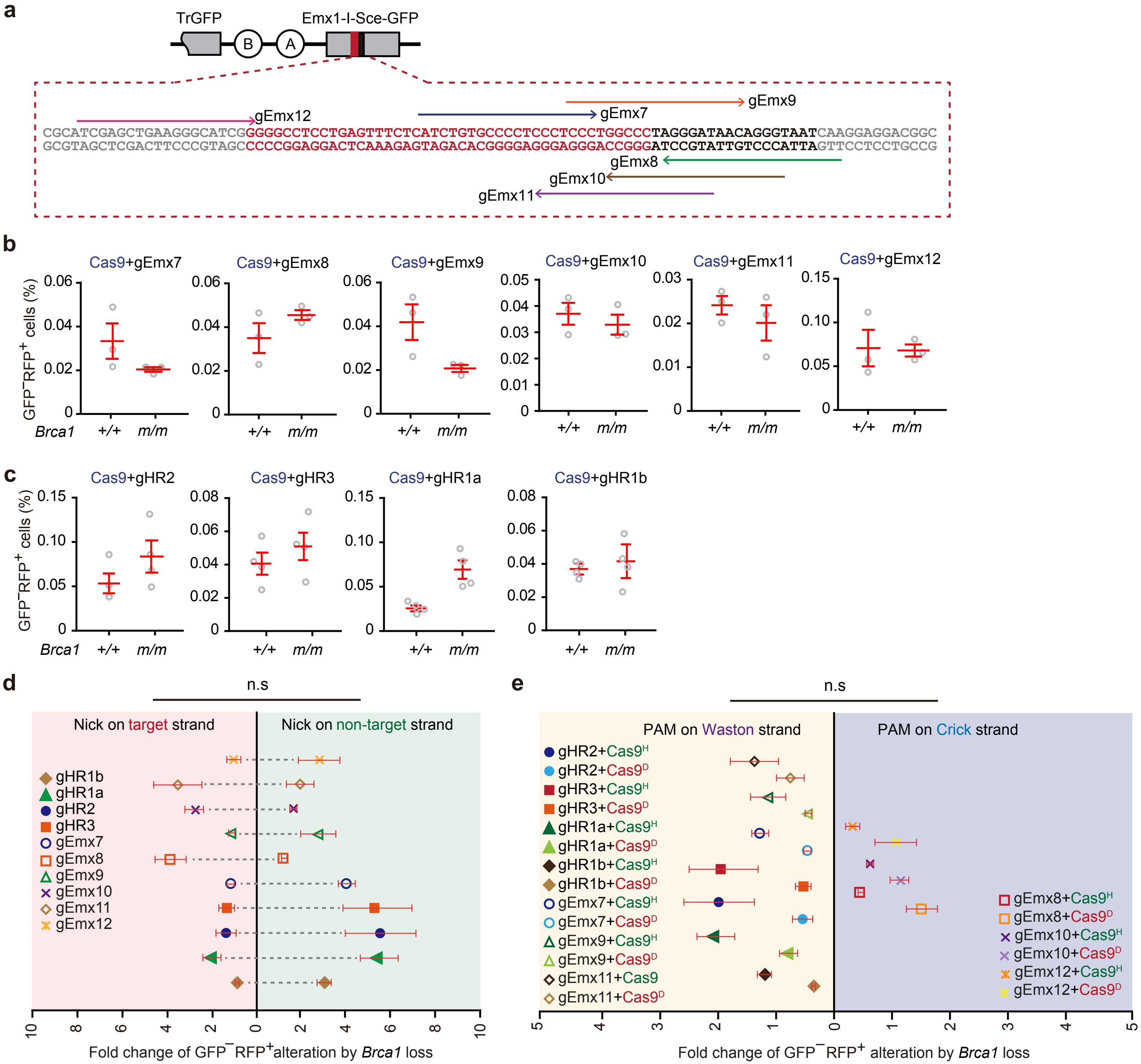
a
TrGFP
B
A
Emx1-I-Sce-GFP
gEmx12
gEmx7
gEmx9
CGCATCGAGCTGAAGGGCATCGGGGGCCTCCTGAGTTTCTCATCTGTGCCCCTCCCTCCCTGGCCCTAGGGATAACAGGGTAATCAAGGAGGACGGC
GCGTAGCTCGACTTCCCGTAGCCCCCGGAGGACTCAAAGAGTAGACACGGGGAGGGAGGGACCGGGATCCCTATTGTCCCATTAGTTCCTCCTGCCG
gEmx8
gEmx10
gEmx11
b
Cas9+gEmx7
Cas9+gEmx8
Cas9+gEmx9
Cas9+gEmx10
Cas9+gEmx11
Cas9+gEmx12
GFP⁻RFP⁺ cells (%)
Brca1
+/+
m/m
c
Cas9+gHR2
Cas9+gHR3
Cas9+gHR1a
Cas9+gHR1b
GFP⁻RFP⁺ cells (%)
Brca1
+/+
m/m
d
n.s
Nick on target strand
Nick on non-target strand
gHR1b
gHR1a
gHR2
gHR3
gEmx7
gEmx8
gEmx9
gEmx10
gEmx11
gEmx12
Fold change of GFP⁻RFP⁺alteration by Brca1 loss
e
n.s
PAM on Waston strand
PAM on Crick strand
gHR2+Cas9H
gHR2+Cas9D
gHR3+Cas9H
gHR3+Cas9D
gHR1a+Cas9H
gHR1a+Cas9D
gHR1b+Cas9H
gHR1b+Cas9D
gEmx7+Cas9H
gEmx7+Cas9D
gEmx9+Cas9H
gEmx9+Cas9D
gEmx11+Cas9
gEmx11+Cas9D
gEmx8+Cas9H
gEmx8+Cas9D
gEmx10+Cas9H
gEmx10+Cas9D
gEmx12+Cas9H
gEmx12+Cas9D
Fold change of GFP⁻RFP⁺ alteration by Brca1 loss

Supplementary Figure 9. Analysis of Cas9-induced GFP^{-}RFP^{+} products in the EMX1-SCR reporter and the SCR-RFP reporter. a Target sequences for 6 sgRNAs around the EMX1-I-SceI site of the EMX1-SCR reporter. Target sequences are indicated by arrowed lines, and the **I**-Sce**I** site and the integrated *EMX1* sequence are respectively in black bold and red bold. **b,c** Frequencies of GFP^{-}RFP^{+} cells induced by Cas9 at 6 independent sites of the EMX1-SCR reporter (**b**) or 4 independent sites of the SCR-RFP reporter (**c**) in mESC. One circle indicates one independent experiment. **d** *Brca1* deficiency-mediated alteration of GFP^{-}RFP^{+} production induced by nicks on the target strand in the DNA-RNA hybrid and on the non-target strand. **e** *Brca1* deficiency-mediated alteration of GFP^{-}RFP^{+} production for nicks induced by Cas9-sgRNA with PAM on the Watson strand and on the Crick strand. Each symbol represents the mean of at least three independent experiments for one sgRNA and two-tailed Student's t-test is performed in d, e. ns, P>0.05.

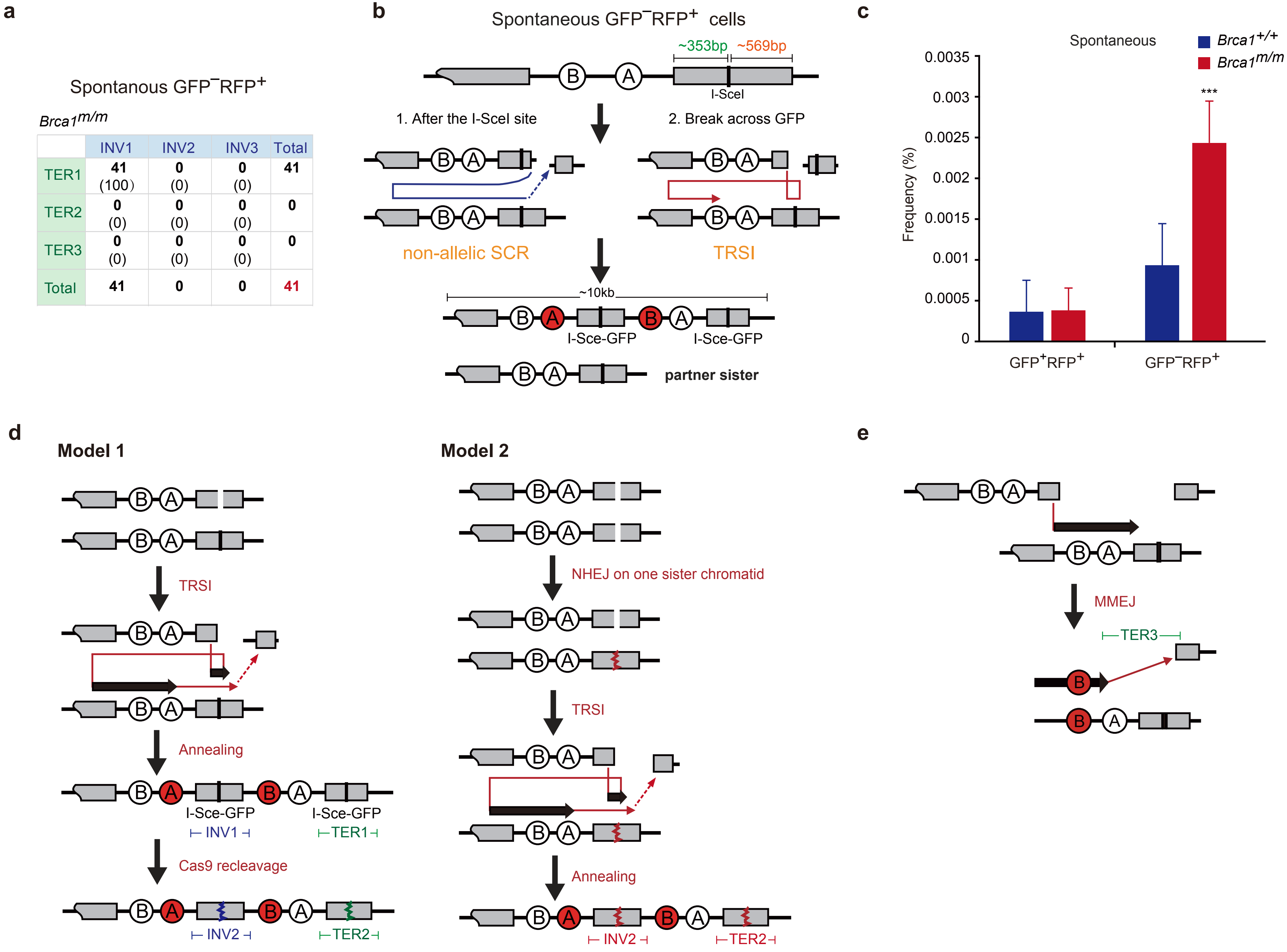

	INV1	INV2	INV3	Total
TER1	41 (100)	0 (0)	0 (0)	41
TER2	0 (0)	0 (0)	0 (0)	0
TER3	0 (0)	0 (0)	0 (0)	0
Total	41	0	0	41

Supplementary Figure 10. Analysis of spontaneous GFP^{-}RFP^{+} cells from the SCR-RFP reporter. a Number (in bold) of spontaneous GFP^{-}RFP^{+} cells with each combined type of invasion and termination junctions in *Brca1$^{m/m}$* cells. The portion of each combined type in GFP^{-}RFP^{+} cells analyzed is shown in parenthesis. **b** Models for spontaneous GFP^{-}RFP^{+} cells. Spontaneous DSBs can occur at 5' (~353 bp) and 3' (~569 bp) side of the I-SceI site in *I-Sce-GFP*. Non-allelic SCR/LTGC (left): Spontaneous break at 3' side of *I-Sce-GFP* can invade non-allelic *TrGFP* to allow TD of the *RFP* exons. TRSI (right): Spontaneous breaks within *I-Sce-GFP* can also invade allelic *I-Sce-GFP* and switch to *TrGFP* for second homologous strand invasion, allowing TD of the *RFP* exons. **c** Frequencies of spontaneous GFP^{+}RFP^{+} and GFP^{-}RFP^{+} cells in *Brca1$^{+/+}$* and *Brca1$^{m/m}$* cells. **d** TRSI-based mechanisms underlying the formation of INV2 and TER2. Model 1: The first invasion into allelic *I-Sce-GFP* restores the intact I-SceI site. After the second invasion at *TrGFP*, the termination of SCR/LTGC by homologous annealing generates the nested *I-Sce-GFP* and a third *I-Sce-GFP*. Cas9 may introduce identical mutations on two *I-Sce-GFP* copies. Model 2: Cas9 mutates *I-Sce-GFP* by NHEJ in the sister chromatid template prior to the first strand invasion. TRSI-mediated TD can generate identical mutations on two *I-Sce-GFP* copies. **e** Mechanism underlying the TER3 formation. Premature displacement of nascent DNA strand before *I-Sce-GFP* during LTGC generates a DNA end for MMEJ with the second end. Thus, TER3 contains the C-terminal half of *GFP*.

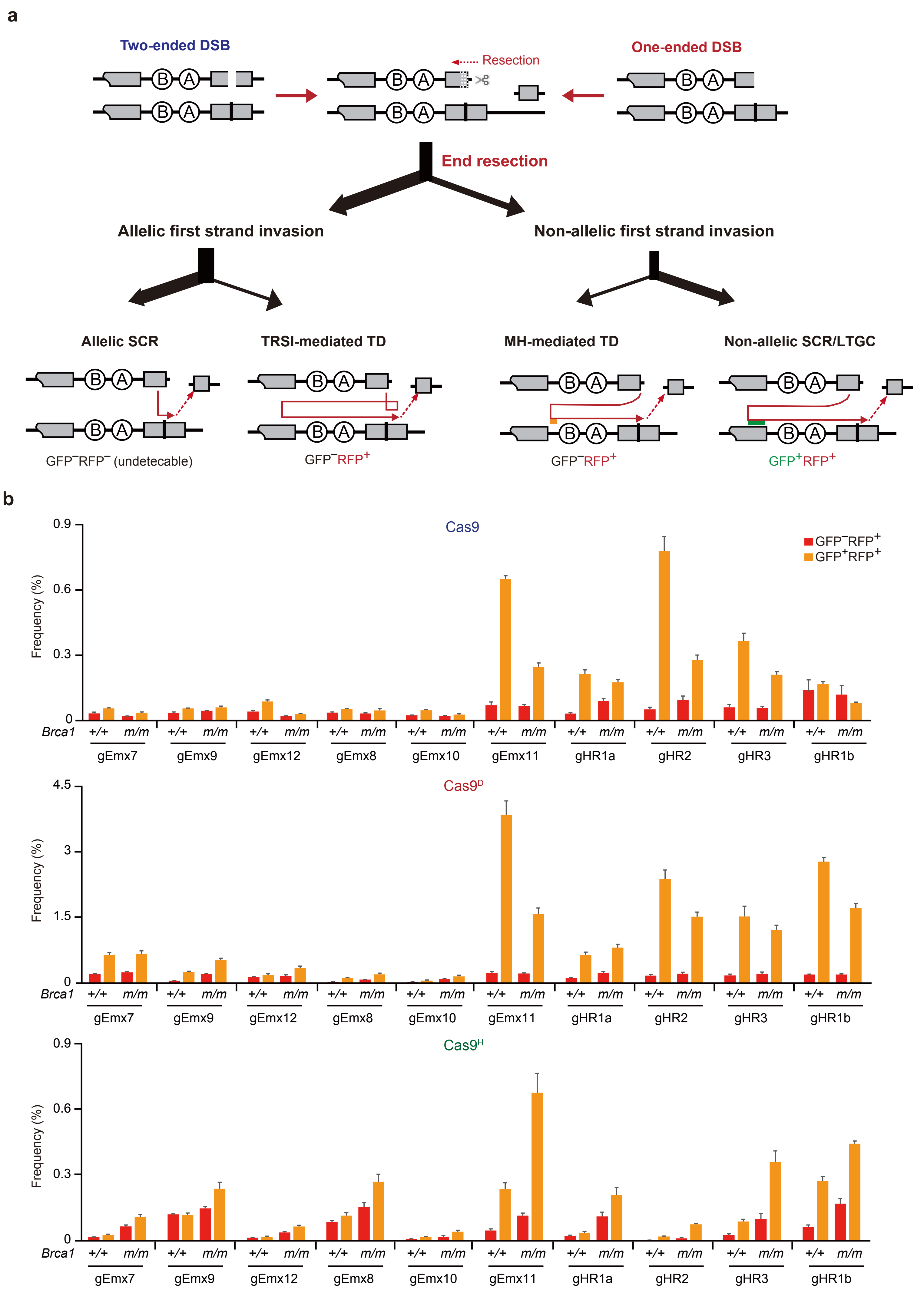

a
Two-ended DSB
Resection
One-ended DSB
End resection
Allelic first strand invasion
Non-allelic first strand invasion
Allelic SCR
TRSI-mediated TD
MH-mediated TD
Non-allelic SCR/LTGC
GFP⁻RFP⁻ (undetecable)
GFP⁻RFP⁺
GFP⁻RFP⁺
GFP⁺RFP⁺
b
Cas9
Cas9D
Cas9H
Frequency (%)
0
0.3
0.6
0.9
1.5
3
4.5
GFP⁻RFP⁺
GFP⁺RFP⁺
Brca1
+/+
m/m
gEmx7
gEmx9
gEmx12
gEmx8
gEmx10
gEmx11
gHR1a
gHR2
gHR3
gHR1b

Supplementary Figure 11. BRCA1 regulates the balance between TDs mediated by non-allelic SCR and TDs mediated by TRSI and MH. a Decision in SCR between several possible TD mechanisms. The choice between first-round allelic strand invasion and non-allelic strand invasion is made after end resection. Allelic strand invasion initiates allelic DNA synthesis for allelic SCR, which is the major recombination mechanism but generally undetectable. Allelic DNA synthesis can also be prematurely terminated to allow second invasion of displaced strand into non-allelic template for TRSI-mediated TDs, which generate $GFP^{-}RFP^{+}$ cells in SCR-RFP reporter cells or EMX1-SCR reporter cells. TDs involving non-allelic first strand invasion include MH-mediated TDs and non-allelic SCR/LTGC if non-allelic homology is present in sister chromatid. MH-mediated TD and non-allelic SCR/LTGC generate $GFP^{-}RFP^{+}$ cells and $GFP^{+}RFP^{+}$ cells in SCR-RFP reporter cells or EMX1-SCR reporter cells, respectively. **b** Different effect of *Brca1* deficiency on frequencies of $GFP^{+}RFP^{+}$ cells and $GFP^{-}RFP^{+}$ cells induced by Cas9 (top), $Cas9^{D}$ (middle) or $Cas9^{H}$ (bottom) at 10 independent sites of the SCR-RFP reporter and the EMX1-SCR reporter in mESC.

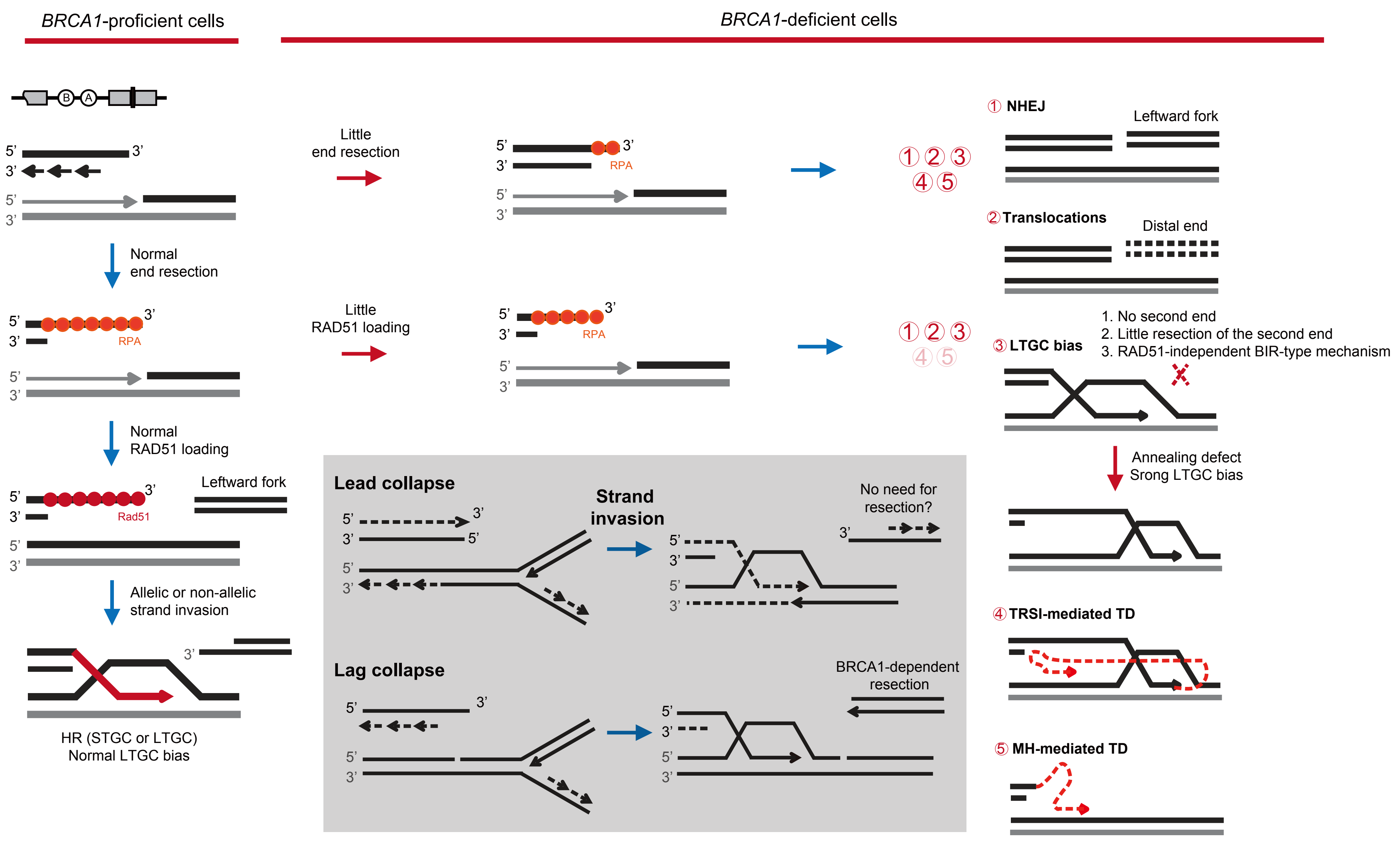

BRCA1-proficient cells
BRCA1-deficient cells
Normal end resection
RPA
Normal RAD51 loading
Rad51
Leftward fork
Allelic or non-allelic strand invasion
HR (STGC or LTGC)
Normal LTGC bias
Little end resection
Little RAD51 loading
① ② ③ ④ ⑤
① NHEJ
Leftward fork
② Translocations
Distal end
③ LTGC bias
1. No second end
2. Little resection of the second end
3. RAD51-independent BIR-type mechanism
Annealing defect
Srong LTGC bias
④ TRSI-mediated TD
⑤ MH-mediated TD
Lead collapse
Strand invasion
No need for resection?
Lag collapse
BRCA1-dependent resection

Supplementary Figure 12. Summary model for the functions of *BRCA1* in repair of one-ended DSBs. In *BRCA1*-proficient cells, one-ended DSBs converted from nicks by DNA replication is repaired by HR with several key steps including end resection, RAD51 loading, strand invasion, DNA synthesis and termination. In *BRCA1*-deficient cells, HR repair of one-ended DSBs is inefficient due to defects in end resection and RAD51 loading, thus promoting alternative repair: ①NHEJ when the second ends are provided by the converging forks; ② Translocation with distal ends; ③LTGC bias increased by limited engagement with the second ends or by the presence of RAD51-independent LTGC-like mechanisms; ④TRSI-mediated TD; and ⑤MH-mediated TD. The faded ④ and ⑤ indicate that the effect of RAD51 loading in these two mechanisms are yet to be determined. In the shaded box: In a lead collapse, the second end generated by the converging fork may contain a long 3' ssDNA tail and require no further resection for strand annealing. In a lag collapse, the second end is likely a blunt end and requires BRCA1-dependent end resection for strand annealing. Thus, the end section function of *BRCA1* at the second ends could induce a strand asymmetry favoring the lag collapse in suppression of LTGC bias.

Supplementary Table 1. sgRNAs used in this study

sgRNA	Sequence (5'-3')	PAM	Target Strand	Length (bp)
gB2(SINE)	GGCTGGAGAGATGGCTCAGG			20
$gBack_W$	ATTCCACACAACATACGAGC	CGG	C	20
$gBack_C$	CTTCCGGCTCGTATGTTGTG	TGG	W	20
gBRCT-2	GGTGCTGATAAAGCAGTGGT	AGG	W	20
gBRCT-4	GACATCTTCAGAAGAAAGAG	CGG	W	20
gHR2	TCGAGCTGAAGGGCATCGTA	GGG	C	20
gHR3	AGGGCATCGTAGGGATAACA	GGG	C	20
gHR1a	AACAGGGTAATCAAGGAGGA	CGG	C	20
gHR1b	GATAACAGGGTAATCAAGG	AGG	C	19
gEMX7	CATCTGTGCCCCTCCCTCCC	TGG	C	20
gEMX8	TTGATTACCCTGTTATCCCT	AGG	W	20
gEMX9	TCCCTGGCCCTAGGGATAAC	AGG	C	20
gEMX10	ACCCTGTTATCCCTAGGGCC	AGG	W	20
gEMX11	TGTTATCCCTAGGGCCAGGG	AGG	W	20
gEMX12	ATCGAGCTGAAGGGCATCGG	GGG	C	20
gEnd-1	GGGCTCTATGGCTTATGAGG	CGG	W	20
gEJ2-2	ATAACAGGGTAATCCATGG	TGG	W	19
gEJ2-3	TGGATTACCCTGTTATCCCT	AGG	C	20
gEJ2-4	ATCCCTAACCGCCGCCACCA	TGG	C	20
gEJ2a	CATGTGAAGGATGGATCCTA	GGG	W	20
gRosa26	CATCCACGCACCCCTGACCC	AGG	C	20

gLDHA	AGCATCACCAAGTGCAGGCA	AGG	C	20
gAlk	TCCTGGCATGTCTATCTGTA	AGG	C	20
gEml4	TTTGTCGGGGTCTACTAGGA	AGG	C	20
gAAVS1	AGACCCAATATCAGGAGACT	AGG	W	20
gEMX1	TCACCTGGGCCAGGGAGGGA	GGG	C	20

Supplementary Table 2. Primers and oligos used in this study

PCR primers	Sequence (5'-3')
BRCT-F	TGGGATCTGGAATCAGCCTT
BRCT-R	GGTACAGAGGACACACTCACT
End-F	GGACGACGGCAACTACAAGA
End-R	CACCTGTTCAATTCCCCTGC
Ldha-F	ATCTATCTCTCCACCCCAATTT
Ldha-R	AGAACACCCATGTGGCAGTT
Rosa26-F	GAGCACAGGAACAATTGGC
Rosa26-R	GGAAAGCCCCTTCTATGCTA
AAVS1-F	CTCCATCGTAAGCAAACC
AAVS1-R	ATATTCCCAGGGCCG
EMX1-F	TATGTAGCCTCAGTCTTCCC
EMX1-R	CTGCTTCGTGGCAATGC
Alk-F	GACCCCAAGAACCTCACTAA
Alk-R	GGTTCACAGATCCAGGGTAT
Gapdh-F	ATGATGCAGTGGCAGGGAGA
Gapdh-R	AATGTGCACGCACCAAGCGT
Eml4-F	AGTTAATCTCAGTAAGAGCC
Eml4-R	ACAGTAAGTTCTAGACCAGC
Inv-F1	GAACGGCCACGAGTTCGAGA
Inv-R1	GGGAGGTGATGTCCAGCTTG
Ter-F1	GGATCTGTGTGGAAAGTCCC
Ter-R1	CGCCAACTAGAGGATCGAGC
EMX1-F	CATCGAGCTGAAGGGCATCGGGGGCCTCCT
EMX1-R	TCGGCCATGATATAGACGTTGTGGCTGTTG
EMX1-oligo	TGTGCCCCAGGATGTTGCCGTCCTCCTTGATTACC<br>CTGTTATCCCTAGGGCCAGGGAGGGAGGGGCACA<br>GATGAGAAACTCAGGAGGCCCCCGATGCCCTTCA<br>GCTCGATGCGGTTCACCAGGGTGTCGCCCTCGAAC
siBRCA1	UCACAGUGUCCUUUAUGUA
qPCR-hBRCA1-F	GGCTATCCTCTCAGAGTGACATTTTA
qPCR-hBRCA1-F	GCTTTATCAGGTTATGTTGCATGGT
qPCR-hUbiquitin-F	CGGCAAGACCATCACTCTGG
qPCR-hUbiquitin-R	AAAGAGTGCGGCCATCTTCC
