# Supplementary material for: DNA nicks induce mutational signatures associated with BRCA1 deficiency
Source: Nat Commun. 2022 Jul 25;13:4285. doi: 10.1038/s41467-022-32011-x (PMC9314409; doi:10.1038/s41467-022-32011-x)
Supplement: Supplementary file 2 — Description of Additional Supplementary Data [file 41467_2022_32011_MOESM2_ESM.pdf]

Description of Additional Supplementary Files

File Name: Supplementary Data 1
Description: Junction analysis of Cas9-gHR3-induced RFP+ cells from BRCA1 mutant cells.
